# Supplementary material for: Collective dynamical regimes predict invasion success and impacts in microbial communities
Source: Nat Ecol Evol. 2025 Jan 6;9(3):406–16. doi: 10.1038/s41559-024-02618-y (PMC11893462; doi:10.1038/s41559-024-02618-y)
Supplement: Supplementary file 1 — Supplementary Materials and Methods, Notes, Discussion, Figs. 1–33 and References. [file 41559_2024_2618_MOESM1_ESM.pdf]

nature ecology & evolution

Supplementary information

https://doi.org/10.1038/s41559-024-02618-y

Collective dynamical regimes predict invasion success and impacts in microbial communities

In the format provided by the authors and unedited

This PDF file includes:

Supplementary Materials and Methods

Supplementary Notes

Supplementary Discussion

Supplementary Figs. 1 to 33

Supplementary References

Supplementary Materials and Methods

Bacterial isolates, media and culturing conditions

We constructed the library of 80 bacterial species from soil, tree leaves, and Charles River water samples taken near the campus of Massachusetts Institute of Technology (Supplementary Fig. 1). This library is phylogenetically diverse, with isolates coming from 24 different families among 5 phylums: Proteobacteria, Firmicutes, Bacteroidota, Actinobacteriota, and Cyanobacteria (Supplementary Fig. 1).

In the case of low interaction strength (low nutrients concentration) conditions, experimental communities were cultured in Base Medium (BM): 1gL^{-1} yeast extract and 1 gL^{-1} soytone from Becton Dickinson, 10 mM sodium phosphate, 0.1 mM CaCl2, 2 mM $MgCl_2$, 4mgL^{-1} $NiSO_4$ and 50 mgL^{-1} $MnCl_2$, pH adjusted to 6.5. For high interaction strength (high nutrients concentration) conditions, we used BM supplemented with 5 gL^{-1} glucose and 4 gL^{-1} urea. We filter sterilized all media using Bottle Top Filtration Units (VWR). All of the chemicals were purchased from Sigma–Aldrich unless otherwise stated.

Both monocultures and communities of the bacterial isolates were grown in 96-deepwell plates (Deepwell plate 96/500µl; Eppendorf) covered with AeraSeal adhesive sealing films (Excel Scientific). The incubation temperature was 30 °C for all communities. We shook the deepwell plates at 1,200 r.p.m. on Titramax shakers (Heidolph). To minimize evaporation, we incubated the plates inside custom-built acrylic boxes.

Pre-cultures, daily dilutions, dispersal, invasion, and biomass measurements

Before each experiment, we initiated the pre-cultures by streak-plating the frozen bacteria on agar plates, picking the single colony, and inoculating individual species into 300 μL of BM. We exposed the resulting monocultures to 5 daily cycles of growth and (30-fold) dilution into fresh media. At the beginning of each experiment, we mixed the aliquots of these monocultures in equal volume proportions to form the synthetic communities. During the experiment, we exposed the monocultures to further dilution cycles and used the monocultures to apply the daily dispersal into the synthetic communities as described below.

We created 40 different synthetic communities using randomly generated subsets of the library of isolates, each subset constituting the species pool (of size S) for each community. After mixing monocultures in equal volumes, each experimental community was initiated by inoculating 10 μL of its initial mix of isolates into 300 μL of BM. We cultured the resulting synthetic communities under serial dilution cycles with dispersal as follows. To apply a 10^{-5} dispersal rate, every 24hr we mixed the monoculture aliquots of the species in each community pool at equal volumes, and then diluted by a 10^3 factor before inoculating 6uL of this mix into the wells containing the corresponding experimental community matching each species pool. After this, we thoroughly mixed the experimental cultures by using a 96-well pipettor (Viaflo 96, Integra Biosciences; settings: pipette/mix program, 5 mixing cycles, mixing volume 150 µL, speed 6) before applying a 30-fold dilution by transferring 10 µL of the cultures into a new plate with 300 µL of fresh media.

We cultured the resident communities over 6 dilution cycles before introducing the invader species. When introducing the invader species on day 6, The volume ratio between the monoculture of invader species and the resident community was 10^{-3}. After introducing the invaders on day 6, we performed another 6 daily dilution cycles with a 10^{-5} dispersal rate for all species including the invader species until the end of the experiment. At the end of every daily cycle, we used 150uL samples of each culture to measure the OD (600nm), a proxy for the total biomass in the cultures, using a Varioskan Flash (Thermo Fisher Scientific) plate reader. The remaining culture volume was stored at -80 °C for subsequent DNA extraction.

We introduced a dispersal rate of 10^{-5} in both of our experiments and gLV simulations. The dispersal from the species pool to the local community is important for maintaining persistent fluctuations in both gLV simulation and our experiment. We found that the lack of dispersal in the gLV model leads to a significantly lower fraction of fluctuating communities (Supplementary Fig. 24). Similarly, the fraction of fluctuating communities is much lower in our experimental communities without daily dispersal (Supplementary Fig. 25). Without dispersal from the species pool, some species reach the extinction boundary due to dramatic fluctuations and cannot recover. This results in a continuous decrease in community fluctuations in both the model and the experiment.

<u>DNA extraction, 16S rRNA sequencing and data analysis.</u>

To monitor the dynamics of the microbial communities, we measured community composition via 16S ribosomal RNA (rRNA) amplicon sequencing. We performed the DNA extraction at the Environmental Sample Preparation and Sequencing Facility at Argonne National Laboratory. We used the obtained DNA to do 16S (V4 region) amplicon sequencing. The Environmental Sample Preparation and Sequencing Facility at Argonne National Laboratory performed the library preparation and Illumina MiSeq sequencing. We used the R package DADA2 to obtain the amplicon sequence variants (ASVs) as described by Callahan *et al.*[57]. We assigned the taxonomic identities to the ASVs by using SILVA (version 132) as a reference database. For each sample, we calculated the species richness as the number of ASVs with a relative abundance ≥0.08%, which corresponds to the 0.08% extinction threshold used in the simulation. We assigned the taxonomic identities to ASVs using Randomized Axelerated Maximum Likelihood (RAxML) using default parameters. In our sequencing dataset, the average sequencing depth is 17075 reads. This means that we could not effectively resolve any species abundance on the order of 0.01% or below. Our main observables, invasion success, diversity and stability, were calculated (Methods) only from species abundances that exceed a threshold of 0.08% (the extinction threshold).

<u>Numerical methods</u>

We modeled the long-term dynamics and diversity of ecological communities using the well-known generalized Lotka-Volterra (gLV) model, modified to include dispersal from a species pool:

$$\frac{dN_i}{dt} = r_i N_i \left(1 - \sum_{j=1}^{S} \alpha_{ij} N_j / K_i\right) + D \qquad (1)$$

where N_i is the abundance of species i (normalized to its carrying capacity), α_{ij} is the interaction strength that captures how strongly species j inhibits the growth of species i (with self-regulation α_{ii} = 1), and D is the dispersal rate from an outside species pool to the focal community. For

simplicity and without qualitatively changing our results, we considered the same growth rate r_i = 1 and the same carrying capacity K_i= 1 for all species in the main text. Our previous paper shows that sampling growth rates from a uniform distribution has little effect on the phase diagram of survival fraction and fluctuation fraction[40]. Our previous paper also shows that sampling carrying capacities from a normal distribution increases the partial coexistence phase while shrinking both the full coexistence phase and fluctuation phase but does not affect the order of the phases[40].

In our previous work, we tested the theoretical predictions when considering the existence of positive (facilitative) interspecies interactions and varying the symmetry of the interaction matrix [40]. We also considered different dispersal rates, and the effects of incorporating daily dilutions in these *in silico* communities[40]. These additional results show that our qualitative phase diagrams and conclusions are robust to different choices of ecological network structure and parameters. Although the patterns of ecological diversity and dynamics do not change as the dispersal rate varies from D=10^{-7} to D=10^{-6}, we found that communities with zero dispersal rate exhibit lower fluctuation fraction and survival faction in the persistent fluctuation phase[40]. Our results showed that non-zero dispersal rates can sustain persistent fluctuations. After the resident species typically reach steady states at t=10^3, we started introducing the invader species by continuously adding dispersal of the invader to the resident community and simulated the dynamics until t=2×10^3 to determine the invasion outcome.

All simulations used the Runge-Kutta method on Matlab to numerically solve the LV equations (with an integration step of 0.05). A definition of 20×20 pixels was used for each phase diagram (Fig. 2f and 4c), linearly segmenting the parameter space in the ranges $<\alpha_{ij}> \in [0.02, 1.1]$ and $S \in [2, 60]$. In each phase diagram, each pixel shows the average result for 10^3 simulations. The total simulation time is 2×10^3. We sampled the interaction strength from a uniform distribution U $[0.5<\alpha_{ij}>, 1.5<\alpha_{ij}>]$ in Fig. 2b and 4d, where $<\alpha_{ij}>$ is the mean interaction strength between species (which also determines here the variance of interactions).

<u>Reaching steady state, extinction threshold, survival fraction and stability in simulations</u>

We define the steady state of simulated communities as the community state in which community properties (e.g., survival fraction, fluctuation fraction, and invasion probability) do not significantly changes as time goes on. To consistently analyze the steady state results for all the simulated communities, in our previous work, we analyzed the dependence of the phase diagrams on the simulated time[40]. Our results showed that community-level properties did not significantly changes after t=10^3. Accordingly, the phase diagrams in the paper show the state of communities at t=2×10^3, unless otherwise stated.

The presence of dispersal from the species pool in Eq. (1) guarantees that all species exhibit strictly positive abundances in Fig 2a. Nevertheless, we consider that a species is extinct if its abundance lays below an 8×10^{-4} threshold, as consistent with the extinction in our experiment. Around this threshold, the dispersal rate becomes the main factor preventing abundance decay[40]. The species abundance distribution in the partial coexistence phase is bimodal[40]; the extinction threshold 8×10^{-4} clearly separates the high-abundance surviving species from low-abundance species that will go extinct if dispersal ceases[40]. We chose the survival threshold for three main reasons: **1) Distinction from dispersal rate**: The survival threshold should be significantly higher than the dispersal rate

to distinguish truly surviving species from those whose low abundance is only sustained by dispersal. Since we used a dispersal rate of 10^{-5}, the threshold should be high enough to be separated from the dispersal floor but not too high to falsely classify surviving species as extinct. Simulation results of gLV suggest that a threshold of 8×10^{-4} efficiently separates surviving species from extinct ones (Supplementary Fig. 26). **2) Sequencing depth**: The sequencing depth is on the order of 10^4. The number of cells transferred in each dilution cycle is on the order of 10^7 cells, and the number of cells used in DNA extraction and amplicon sequencing is on the order of 10^6 cells. Therefore, the detection limit in our sequencing data is primarily determined by the sequencing depth of each community. Any threshold below the order of 10^{-4} would be inconsistent with the detection limit of our sequencing depth. We chose 8×10^{-4}, which is sufficiently above the detection limit but not too high to exclude low-abundance surviving species. **3) Robustness of conclusion**: We found that varying the survival threshold between 10^{-4} and 10^{-3} does not alter our key conclusions. Specifically, a survival threshold of 10^{-4} yielded exactly the same number of successful invasions as a threshold of 8×10^{-4} in our experiment. The survival threshold of 10^{-3} resulted in 61 successful invasions out of 244 total invasion tests, compared to 63 successful invasions out of 244 total invasion tests under the 8×10^{-4} threshold. There were only two cases where the invader abundance fell between 8×10^{-4} and 10^{-3}, leading to a minor quantitative difference in the number of successful invasions. We further verified that this small difference does not affect our major conclusions, including (1) that fluctuating communities are more invasible than stable communities, and (2) that invasion probability decreases with increasing species pool and interaction strength.

To compute the survival fraction, we computed the fraction of species whose abundance exceeded the extinction threshold at any time during the last 100 units of time in the simulation. The survival or extinction of invader species was determined by the abundance in the last 100 units of time in the simulation. Our choice of including a time window when measuring diversity is motivated by the fact that, for the case of unstable communities, species abundances fluctuate above and below the extinction threshold over time. Since we measured diversity and species compositions every 24 hours in the experiment, we consider an analogous window of 100 time units in simulations.

To differentiate between stable and fluctuating communities, we calculated the maximal coefficient of variation (CV) of N_i across all species in a community during the time window from $t=10^3-100$ to $t=10^3$. We define a community as fluctuating if this maximal CV exceeds 10^{-3}, and as stable if it falls below this threshold. Fluctuating communities are characterized by relatively large maximum CV values, while stable communities exhibit small maximum CV values, leading to a bimodal distribution. The threshold of 10^{-3} effectively serves as a valley threshold to distinguish between these two peaks.

Definition of stable and fluctuating experimental communities

To differentiate between stable and fluctuating resident communities in experiments, we computed the standard deviation of biomass between day 4, day 5 and day 6. Communities for which the standard deviation of biomass over time is below (above) a 0.05 threshold are considered stable (fluctuating) communities (Supplementary Fig. 12). We also calculated the average coefficient of variation (CV) for species abundances from day 4 to day 6. This corresponds to the average value of the standard deviation for the absolute abundance of each species N_i (over day 4, day 5, and day

6) scaled by average species abundance. The average coefficient of variation of absolute species abundance (product of biomass and relative species abundance by 16s sequencing) displays a strong positive correlation with the standard deviation of biomass over time across communities (Supplementary Fig. 12). The average coefficient of variation of relative species abundance (by 16s sequencing) also displays a strong positive correlation with the standard deviation of biomass over time across communities (Supplementary Fig. 12). Different metrics consistently classify the communities into two clusters: fluctuating ones on top right region and stable ones on bottom left region (Supplementary Fig. 12). Varying the choice of time window (day 4 to day 6) to a new time window (day 3 to day 6) yield the same classification of fluctuating and stable communities. Our previous work showed that the classification of stability converges quickly to either small or large values, respectively indicating stability or long-lasting fluctuations in experimental communities[40]. We found the *K*-means clustering algorithm yields the same classification results of community stability. The consistence between results of *K*-means clustering and setting stability threshold of biomass standard deviation (0.05) demonstrates the classification of fluctuating and stable communities is robust to different algorithms.

In this study, all fluctuations are referred to as deterministic fluctuations (chaos or limit cycle oscillations) driven by inter-species interactions, rather than stochastic fluctuations driven by demographic noise. The reason we believe our experiment is in the deterministic population dynamics regime, rather than the stochastic regime, is due to the large population size in our experimental communities. Colony plating and counting in our experiment indicate that the CFU is on the order of 10^9 per mL. We cultured each community in a 300 μL medium, consisting of around 3×10^8 cells, and transferred 10 μL of the community cultures into a new plate with 300 μL of fresh media in each dilution cycle, transferring about 10^7 cells. The relative ratio of deterministic population growth to stochastic birth and death is on the order of $1/\sqrt{n}$, which is very low given the total population size of 10^7 to 3×10^8 cells in our experiment. Additionally, our communities display stable species composition in stable communities, with sequencing results showing that species composition does not exhibit significant stochastic fluctuations[40]. This suggests that stochastic fluctuations play a less important role in our experiment.

Supplementary Notes

Theoretical alternatives to the Lotka-Volterra model

To directly model the pH-mediated interactions in our framework, we consider a minimal model where the pH value (p in the model) influences the species' per capita growth rates linearly, and, reciprocally, the species modify the environmental pH in a linear manner. This approach serves as a straightforward extension of the generalized Lotka-Volterra (gLV) model, which traditionally handles species interactions without considering environmental feedback such as pH.

In this extended model, the interaction between species and pH is twofold: species can alter the pH of their environment, and this altered pH, in turn, affects their growth rates. This dual influence allows the model to capture more complex ecological dynamics, where pH acts as a mediating factor that can shift the balance between competition, cooperation, and exploitation among species. Depending on the specific parameters chosen—such as the sensitivity of growth rates to pH

changes and the extent to which species modify pH—the model can simulate a variety of interaction scenarios.

For instance, a species that raises the environmental pH could either inhibit or promote the growth of other species, depending on whether those species thrive in higher or lower pH conditions. Similarly, species that lower the pH could create environments that are hostile or favorable to others, depending on their pH preferences. This dynamic allows the model to represent competitive interactions (where species indirectly harm each other by altering pH), cooperative interactions (where species create favorable conditions for each other), and exploitative interactions (where one species benefits at the expense of another by altering the pH).

By incorporating pH-mediated interactions, this model adds a layer of realism to the gLV framework, making it more applicable to ecosystems where environmental factors like pH play a critical role in shaping species interactions. The linear relationships assumed in this model are not just mathematically convenient; they also provide a minimal yet powerful way to explore how environmental feedback can influence community dynamics. This extension allows for the modeling of a broader range of ecological scenarios, potentially offering new insights into how species coexist and compete in pH-sensitive environments.

$$\boldsymbol{\frac{dN_i}{dt} = N_i\left(1 - N_i - \sum_j \alpha_{ij}N_j + g_i p\right)} \quad (2)$$

$$\boldsymbol{\dot{p} = -\delta p + \beta \sum_j k_j N_j} \quad (3)$$

In the adiabatic limit, where the rate of pH change is much faster than the species' growth rates (time scale separation), or at steady state, we can assume $\dot{p} = -\delta p + \beta \sum_j k_j N_j = 0$. This implies that $p \cong \beta^* \sum k_j N_j$, where $\beta^* = \beta/\delta$. Substituting $p \cong \beta^* \sum k_j N_j$ into equation (2), we obtained:

$$\boldsymbol{\frac{dN_i}{dt} = N_i\left(1 - \frac{N_i + \sum \alpha_{ij}^* N_j}{K_i^*}\right)} \quad (4)$$

where the effective gLV-type interaction strength is given by $\alpha_{ij}^* = \frac{(\alpha_{ij} - \beta^* g_i k_j)}{(1 - \beta^* g_i k_i)}$, and the effective carrying capacity can be calculated through $\frac{1}{K_i^*} = (1 - \beta^* g_i k_i)$. Here, g_i quantifies how pH influences species growth, while k_j quantifies how species alter the environmental pH. The parameter δ represents the recovery rate of pH due to the addition of fresh medium at the end of each serial dilution cycle (with pH being neutral, or 7, after each addition). In the model, p=0 corresponds to neutral pH = 7 in the experiment, p>0 and p<0 represents alkaline and acidic pH respectively. The parameter β represents the environmental coupling strength, which quantifies the impact of species on environmental pH and reflects the phosphate buffering concentration in the experiment. The normalized environmental coupling strength, considering the competing effects of pH recovery (δ) and species-induced pH changes (determined by β), is given by $\beta^* = \beta/\delta$.

These results demonstrate that pH-mediated interactions can be effectively incorporated into the gLV framework. The compatibility of the gLV model with pH-mediated interactions justifies our

choice, as it allows us to account for environmental factors like pH without sacrificing the simplicity and analytical power of the gLV model.

To further explore the pH model's predictions on invasion outcomes, we simulated the model combining both pH and gLV interaction effects across various parameter spaces. We found that even when considering only the pH effect, the model predicts a decrease in invasion probability with increasing pH interaction strength (mean of $\beta^* g_i k_i$) and species pool size (Supplementary Fig. 23), which qualitatively aligns with the predictions of the pure gLV model (Fig. 2f). In this purely pH-mediated interaction regime, the survival fraction is approximately equal to the invasion probability (Supplementary Fig. 23).

Moreover, when both gLV interaction strength (mean of α_{ij}) and pH-mediated interaction strength (mean of $\beta^* g_i k_i$) are increased simultaneously, the invasion probability decreases (Supplementary Fig. 23) in a manner similar to that predicted by the gLV model alone (Fig. 2f). In all conditions, the survival fraction remains an efficient predictor of invasion probability (Supplementary Fig. 23), as observed in the pure gLV model (Fig. 4).

These results demonstrate that the dependence of invasion probability on interaction strength, species pool size, and survival fraction is robust across both pure pH and combined pH-gLV models. This further justifies our choice of the gLV model, as it successfully predicts key features of invasion behavior in communities. By incorporating pH-mediated interactions into the gLV framework, we capture the added complexity of pH effects while retaining the simplicity and analytical power of the gLV model.

<u>Justification for the choice of the Lotka-Volterra model</u>

As a phenomenological model, generalized Lotka-Volterra (gLV) model describes species interactions through a pair-wise interaction matrix, capturing how the abundance of one species influences the growth of others. The interaction matrix in the gLV framework is a high-level description that can incorporate various mechanisms, including resource competition, cross-protection, cross-toxin, cross-feeding, and pH-mediated interactions.

It is important to emphasize that the pH-mediated interaction is not the only sole mechanism in our experiment. Some communities show fluctuating biomass while maintaining stable pH levels, indicating other mechanisms, such as cross-toxin interactions, are at play, particularly under high nutrient concentrations. Furthermore, our model considering only pH-mediated interactions cannot explain all experimental observations. For instance, our experiments and gLV simulations show fluctuating communities with higher diversity and invasibility [40], while our pH-only models predict lower diversity and invasibility (Supplementary Fig. 10-11 of Hu et al. 2022)[40], contrary to our experimental findings. These justifications support our use of the gLV model and align with recent progress in using phenomenological models for demographic manipulations[58].

To further justify our modelling approach, where increasing nutrient levels in the experiment correspond to corresponds to amplifying average interaction strength in the gLV model, we conducted pairwise coculture experiments. These experiments revealed that as nutrient concentration increased, the fraction of species pairs that coexisted decreased, while the fraction

of competitive exclusions (where only one species survives) increased[40], reflecting the increase in interspecies interaction strength within the framework of the gLV model.

Supplementary Discussion

Invasion effect

To demonstrate that the invasion causes a statistically significant effect on community structure under high nutrient conditions, we performed a one-sample t-test on the invasion effect data shown in Fig. 5d (right panel, under high nutrient conditions). The results indicate that the invasion effect is significantly greater than zero ($p = 1.11 \times 10^{-6}$). The invasion effect is measured as the proportion of change in surviving species before the invasion (on day 6) and after the invasion (on day 12), calculated through: 1 - (number of overlapping species / total number of species). To demonstrate that the invasion causes a statistically significant effect on community structure under low nutrient conditions, we performed a one-sample t-test on the invasion effect data shown in Fig. 5d (left panel, under low nutrient conditions). The results indicate that the invasion effect is significantly greater than zero ($p = 2.07 \times 10^{-25}$).

Priority effect and alternative stable states

Despite the experimentally observed correspondence between invasion probability and survival fraction, we note that the invasion probability for communities under high nutrient (strong interaction) conditions is usually lower than their survival fraction (i.e. the majority of points on the bottom left are below the diagonal line on Fig. 3c). We now discuss how this can be interpreted in terms of priority effects or alternative stable states. If the assembly of species in the community does not depend on the order of species arrival (i.e., no effect of history), the survival fraction of species assembled from the initial pool should be statistically equal to the survival probability of the species that invade the communities later. In ecology, a "priority effect" refers to a situation in which the community structure is influenced by the order and timing of species' arrival[44,45]. We thus interpret the mismatch between invasion probability and survival fraction under high nutrient concentrations (strong interactions) as evidence of priority effects in the community assembly under strong interactions in our experiment. Early-arriving species can dominate by making it more challenging for subsequent invaders to establish, leading to a lower invasion probability than the survival fraction of species in the original pool. Under weak interactions, however, the colonization probability of invader species is similar to the probability of a species in the initial pool surviving the process of community assembly(Fig. 3c)[10]. An emergent priority effect in communities composed of strongly interacting species can be explained by the presence of alternative stable states with different species compositions, which we observe in additional experiments (Supplementary Fig. 22). Early-arriving species establish dominance, making it more difficult for later-arriving invaders to successfully establish themselves, by inhibiting their growth at low abundance. These alternate stable states thus explain why the invasion probability is generally lower than the survival fraction from the initial pool under high nutrient (strong interaction) conditions[10,40].

Supplementary Figures

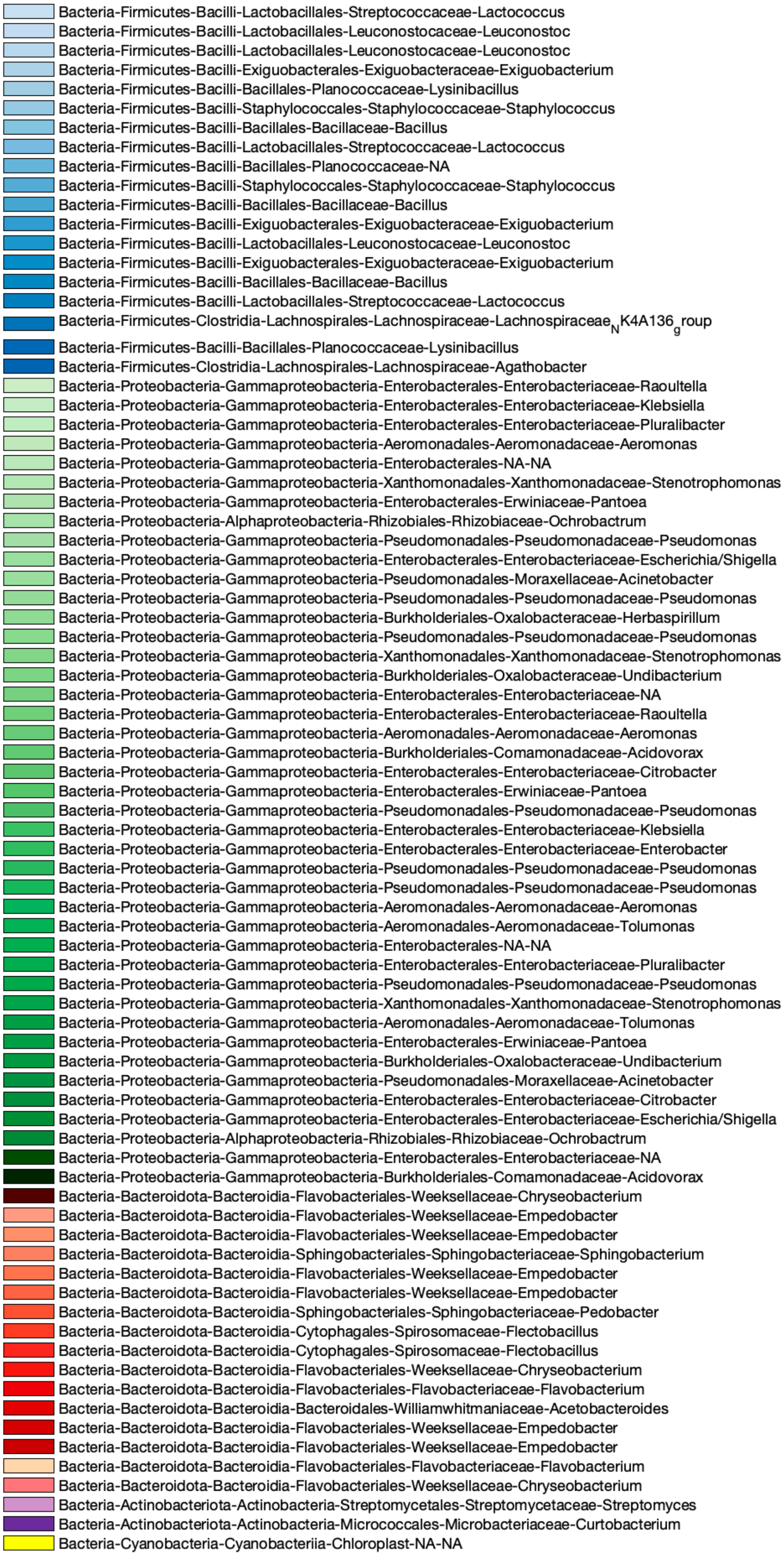

Bacteria-Firmicutes-Bacilli-Lactobacillales-Streptococcaceae-Lactococcus
Bacteria-Firmicutes-Bacilli-Lactobacillales-Leuconostocaceae-Leuconostoc
Bacteria-Firmicutes-Bacilli-Lactobacillales-Leuconostocaceae-Leuconostoc
Bacteria-Firmicutes-Bacilli-Exiguobacterales-Exiguobacteraceae-Exiguobacterium
Bacteria-Firmicutes-Bacilli-Bacillales-Planococcaceae-Lysinibacillus
Bacteria-Firmicutes-Bacilli-Staphylococcales-Staphylococcaceae-Staphylococcus
Bacteria-Firmicutes-Bacilli-Bacillales-Bacillaceae-Bacillus
Bacteria-Firmicutes-Bacilli-Lactobacillales-Streptococcaceae-Lactococcus
Bacteria-Firmicutes-Bacilli-Bacillales-Planococcaceae-NA
Bacteria-Firmicutes-Bacilli-Staphylococcales-Staphylococcaceae-Staphylococcus
Bacteria-Firmicutes-Bacilli-Bacillales-Bacillaceae-Bacillus
Bacteria-Firmicutes-Bacilli-Exiguobacterales-Exiguobacteraceae-Exiguobacterium
Bacteria-Firmicutes-Bacilli-Lactobacillales-Leuconostocaceae-Leuconostoc
Bacteria-Firmicutes-Bacilli-Exiguobacterales-Exiguobacteraceae-Exiguobacterium
Bacteria-Firmicutes-Bacilli-Bacillales-Bacillaceae-Bacillus
Bacteria-Firmicutes-Bacilli-Lactobacillales-Streptococcaceae-Lactococcus
Bacteria-Firmicutes-Clostridia-Lachnospirales-Lachnospiraceae-Lachnospiraceae$_N$K4A136$_g$roup
Bacteria-Firmicutes-Bacilli-Bacillales-Planococcaceae-Lysinibacillus
Bacteria-Firmicutes-Clostridia-Lachnospirales-Lachnospiraceae-Agathobacter
Bacteria-Proteobacteria-Gammaproteobacteria-Enterobacterales-Enterobacteriaceae-Raoultella
Bacteria-Proteobacteria-Gammaproteobacteria-Enterobacterales-Enterobacteriaceae-Klebsiella
Bacteria-Proteobacteria-Gammaproteobacteria-Enterobacterales-Enterobacteriaceae-Pluralibacter
Bacteria-Proteobacteria-Gammaproteobacteria-Aeromonadales-Aeromonadaceae-Aeromonas
Bacteria-Proteobacteria-Gammaproteobacteria-Enterobacterales-NA-NA
Bacteria-Proteobacteria-Gammaproteobacteria-Xanthomonadales-Xanthomonadaceae-Stenotrophomonas
Bacteria-Proteobacteria-Gammaproteobacteria-Enterobacterales-Erwiniaceae-Pantoea
Bacteria-Proteobacteria-Alphaproteobacteria-Rhizobiales-Rhizobiaceae-Ochrobactrum
Bacteria-Proteobacteria-Gammaproteobacteria-Pseudomonadales-Pseudomonadaceae-Pseudomonas
Bacteria-Proteobacteria-Gammaproteobacteria-Enterobacterales-Enterobacteriaceae-Escherichia/Shigella
Bacteria-Proteobacteria-Gammaproteobacteria-Pseudomonadales-Moraxellaceae-Acinetobacter
Bacteria-Proteobacteria-Gammaproteobacteria-Pseudomonadales-Pseudomonadaceae-Pseudomonas
Bacteria-Proteobacteria-Gammaproteobacteria-Burkholderiales-Oxalobacteraceae-Herbaspirillum
Bacteria-Proteobacteria-Gammaproteobacteria-Pseudomonadales-Pseudomonadaceae-Pseudomonas
Bacteria-Proteobacteria-Gammaproteobacteria-Xanthomonadales-Xanthomonadaceae-Stenotrophomonas
Bacteria-Proteobacteria-Gammaproteobacteria-Burkholderiales-Oxalobacteraceae-Undibacterium
Bacteria-Proteobacteria-Gammaproteobacteria-Enterobacterales-Enterobacteriaceae-NA
Bacteria-Proteobacteria-Gammaproteobacteria-Enterobacterales-Enterobacteriaceae-Raoultella
Bacteria-Proteobacteria-Gammaproteobacteria-Aeromonadales-Aeromonadaceae-Aeromonas
Bacteria-Proteobacteria-Gammaproteobacteria-Burkholderiales-Comamonadaceae-Acidovorax
Bacteria-Proteobacteria-Gammaproteobacteria-Enterobacterales-Enterobacteriaceae-Citrobacter
Bacteria-Proteobacteria-Gammaproteobacteria-Enterobacterales-Erwiniaceae-Pantoea
Bacteria-Proteobacteria-Gammaproteobacteria-Pseudomonadales-Pseudomonadaceae-Pseudomonas
Bacteria-Proteobacteria-Gammaproteobacteria-Enterobacterales-Enterobacteriaceae-Klebsiella
Bacteria-Proteobacteria-Gammaproteobacteria-Enterobacterales-Enterobacteriaceae-Enterobacter
Bacteria-Proteobacteria-Gammaproteobacteria-Pseudomonadales-Pseudomonadaceae-Pseudomonas
Bacteria-Proteobacteria-Gammaproteobacteria-Pseudomonadales-Pseudomonadaceae-Pseudomonas
Bacteria-Proteobacteria-Gammaproteobacteria-Aeromonadales-Aeromonadaceae-Aeromonas
Bacteria-Proteobacteria-Gammaproteobacteria-Aeromonadales-Aeromonadaceae-Tolumonas
Bacteria-Proteobacteria-Gammaproteobacteria-Enterobacterales-NA-NA
Bacteria-Proteobacteria-Gammaproteobacteria-Enterobacterales-Enterobacteriaceae-Pluralibacter
Bacteria-Proteobacteria-Gammaproteobacteria-Pseudomonadales-Pseudomonadaceae-Pseudomonas
Bacteria-Proteobacteria-Gammaproteobacteria-Xanthomonadales-Xanthomonadaceae-Stenotrophomonas
Bacteria-Proteobacteria-Gammaproteobacteria-Aeromonadales-Aeromonadaceae-Tolumonas
Bacteria-Proteobacteria-Gammaproteobacteria-Enterobacterales-Erwiniaceae-Pantoea
Bacteria-Proteobacteria-Gammaproteobacteria-Burkholderiales-Oxalobacteraceae-Undibacterium
Bacteria-Proteobacteria-Gammaproteobacteria-Pseudomonadales-Moraxellaceae-Acinetobacter
Bacteria-Proteobacteria-Gammaproteobacteria-Enterobacterales-Enterobacteriaceae-Citrobacter
Bacteria-Proteobacteria-Gammaproteobacteria-Enterobacterales-Enterobacteriaceae-Escherichia/Shigella
Bacteria-Proteobacteria-Alphaproteobacteria-Rhizobiales-Rhizobiaceae-Ochrobactrum
Bacteria-Proteobacteria-Gammaproteobacteria-Enterobacterales-Enterobacteriaceae-NA
Bacteria-Proteobacteria-Gammaproteobacteria-Burkholderiales-Comamonadaceae-Acidovorax
Bacteria-Bacteroidota-Bacteroidia-Flavobacteriales-Weeksellaceae-Chryseobacterium
Bacteria-Bacteroidota-Bacteroidia-Flavobacteriales-Weeksellaceae-Empedobacter
Bacteria-Bacteroidota-Bacteroidia-Flavobacteriales-Weeksellaceae-Empedobacter
Bacteria-Bacteroidota-Bacteroidia-Sphingobacteriales-Sphingobacteriaceae-Sphingobacterium
Bacteria-Bacteroidota-Bacteroidia-Flavobacteriales-Weeksellaceae-Empedobacter
Bacteria-Bacteroidota-Bacteroidia-Flavobacteriales-Weeksellaceae-Empedobacter
Bacteria-Bacteroidota-Bacteroidia-Sphingobacteriales-Sphingobacteriaceae-Pedobacter
Bacteria-Bacteroidota-Bacteroidia-Cytophagales-Spirosomaceae-Flectobacillus
Bacteria-Bacteroidota-Bacteroidia-Cytophagales-Spirosomaceae-Flectobacillus
Bacteria-Bacteroidota-Bacteroidia-Flavobacteriales-Weeksellaceae-Chryseobacterium
Bacteria-Bacteroidota-Bacteroidia-Flavobacteriales-Flavobacteriaceae-Flavobacterium
Bacteria-Bacteroidota-Bacteroidia-Bacteroidales-Williamwhitmaniaceae-Acetobacteroides
Bacteria-Bacteroidota-Bacteroidia-Flavobacteriales-Weeksellaceae-Empedobacter
Bacteria-Bacteroidota-Bacteroidia-Flavobacteriales-Weeksellaceae-Empedobacter
Bacteria-Bacteroidota-Bacteroidia-Flavobacteriales-Flavobacteriaceae-Flavobacterium
Bacteria-Bacteroidota-Bacteroidia-Flavobacteriales-Weeksellaceae-Chryseobacterium
Bacteria-Actinobacteriota-Actinobacteria-Streptomycetales-Streptomycetaceae-Streptomyces
Bacteria-Actinobacteriota-Actinobacteria-Micrococcales-Microbacteriaceae-Curtobacterium
Bacteria-Cyanobacteria-Cyanobacteriia-Chloroplast-NA-NA

Supplementary Fig. 1. Taxonomic identity of the bacterial isolates. The identities have been inferred from the ASV (Methods) of 16S sequencing, which allow the classification of the 80 isolates down to the genus level. Colors are consistent with those in the main text and other supplementary figures. Species belonging to the Firmicutes phylum are assigned different shades of blue, Proteobacteria species are assigned different shades of green, Bacteroidota species are assigned different shades of red, Actinobacteriota species are assigned different shades of purple, Cyanobacteria species are assigned different shades of yellow.

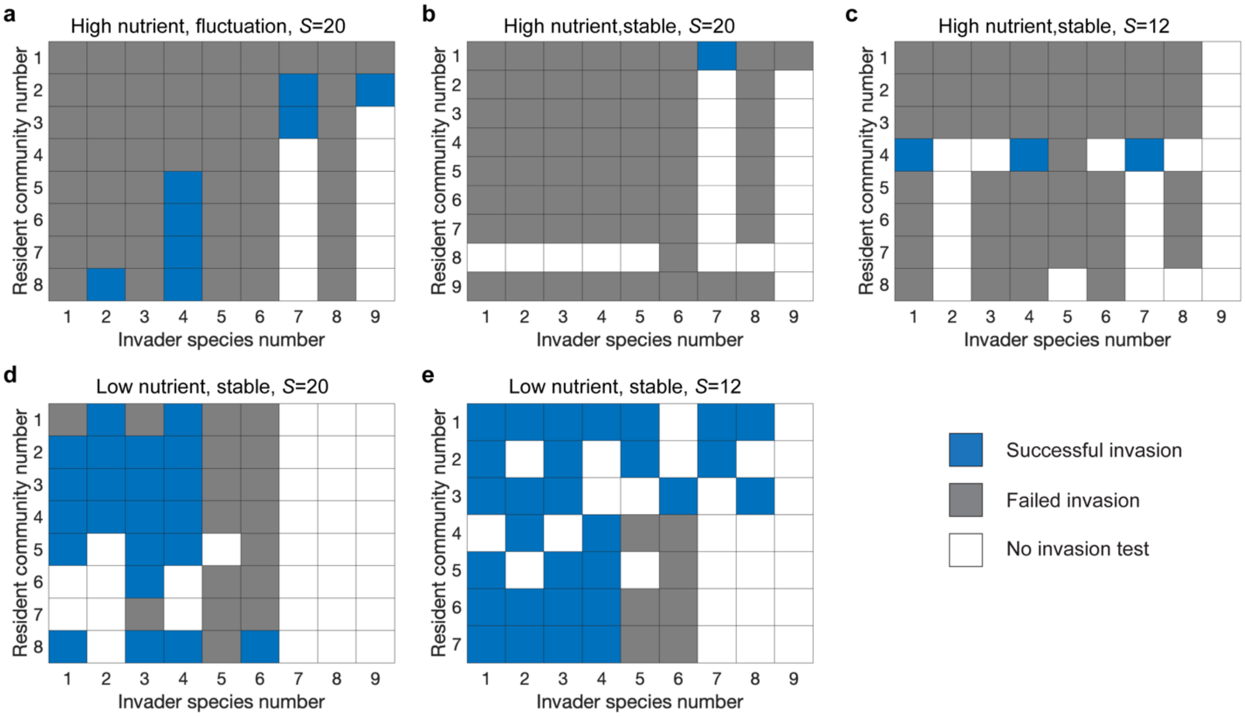

Supplementary Fig. 2. Introducing different invaders into different resident communities and measuring the invasion outcome through 16s sequencing. The invasion outcome matrices show that increasing nutrient and species pool size lead to a decrease in invasion probability.

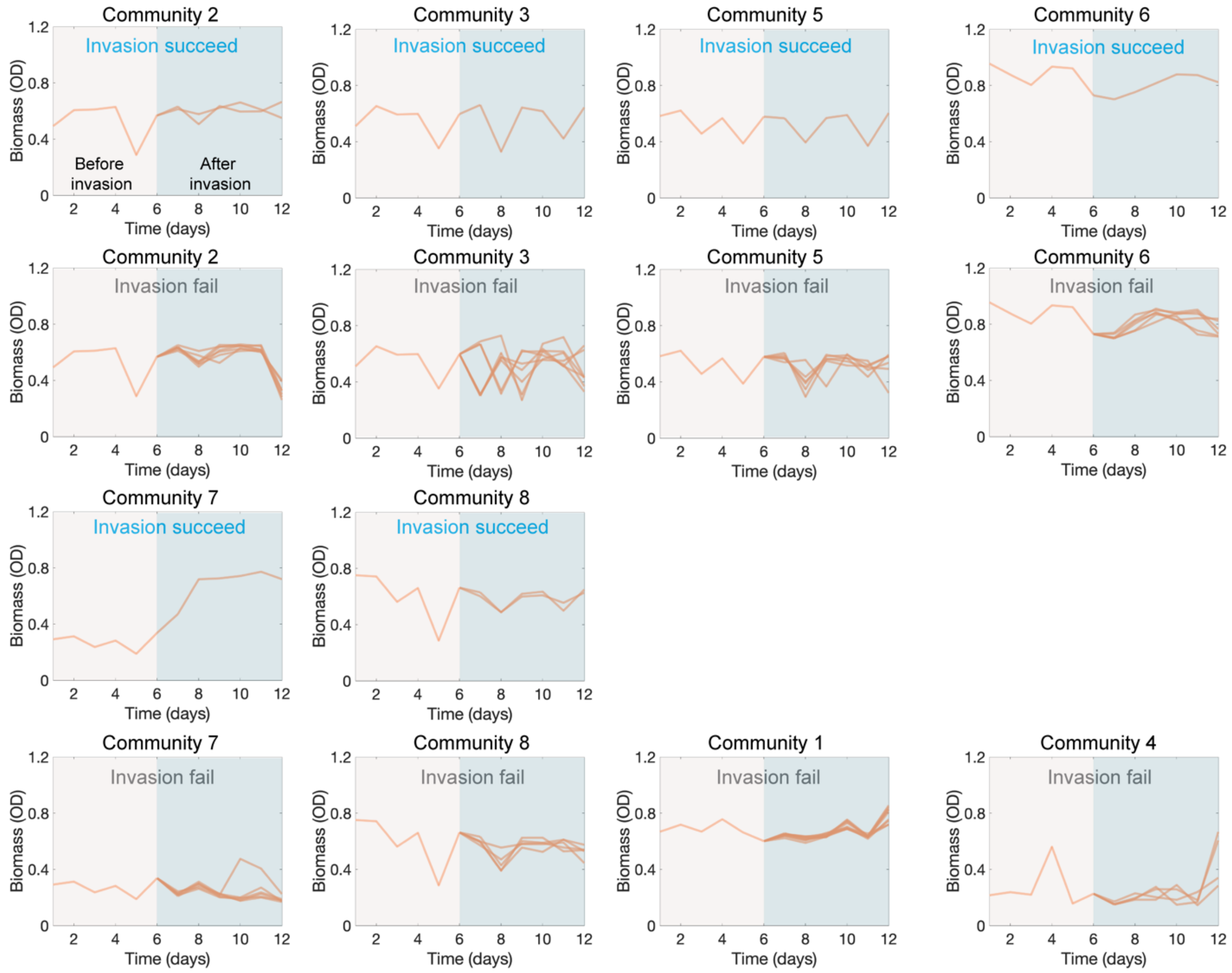

Supplementary Fig. 3. Time series for the biomass of the fluctuating communities with species pool size *S*=20 under strong average interaction strength (high nutrients concentration). Each panel shows the time series for the OD (600nm) of one fluctuating community with species pool size *S*=20 under high nutrient. The invaders were introduced on day 6, and the time series of successful invasions and failed invasions for the same communities were displayed in different panels.

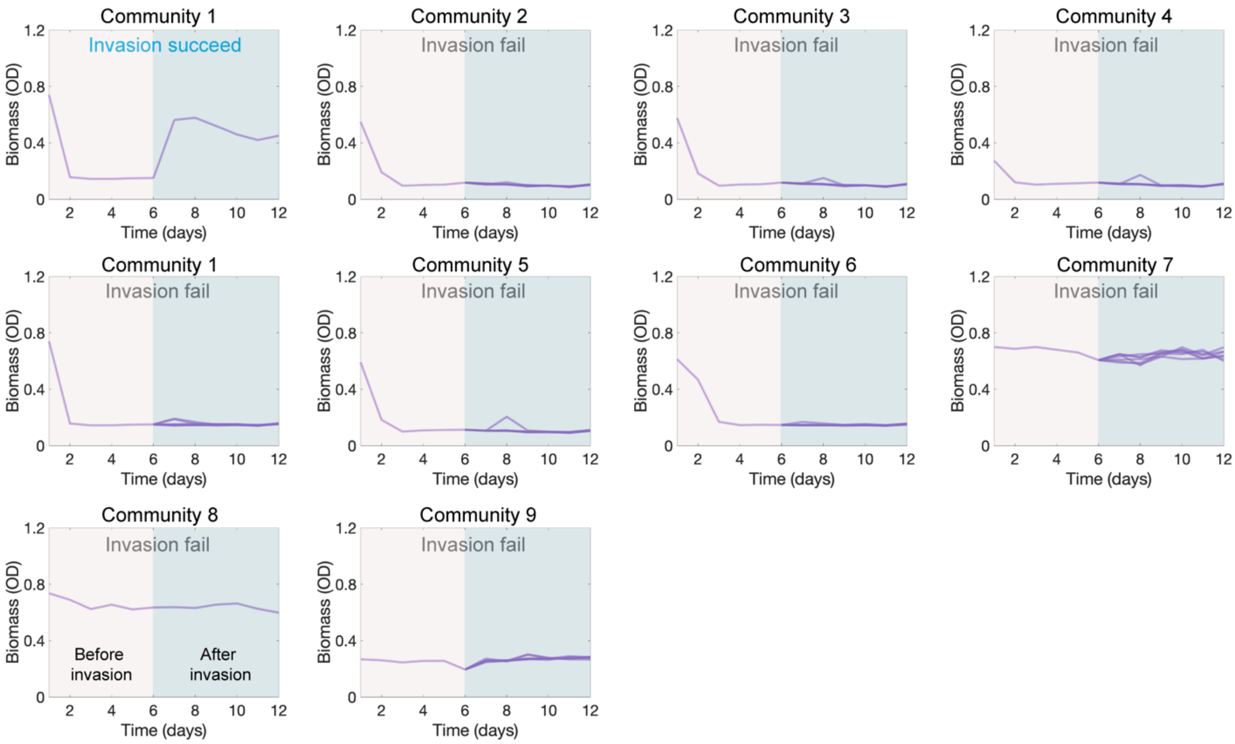

Supplementary Fig. 4. Time series for the biomass of the stable communities with species pool size *S*=20 under strong average interaction strength (high nutrients concentration). Each panel shows the time series for the OD (600nm) of one stable community with species pool size *S*=20 under high nutrient. The invaders were introduced on day 6, and the time series of successful invasions and failed invasions for the same communities were displayed in different panels.

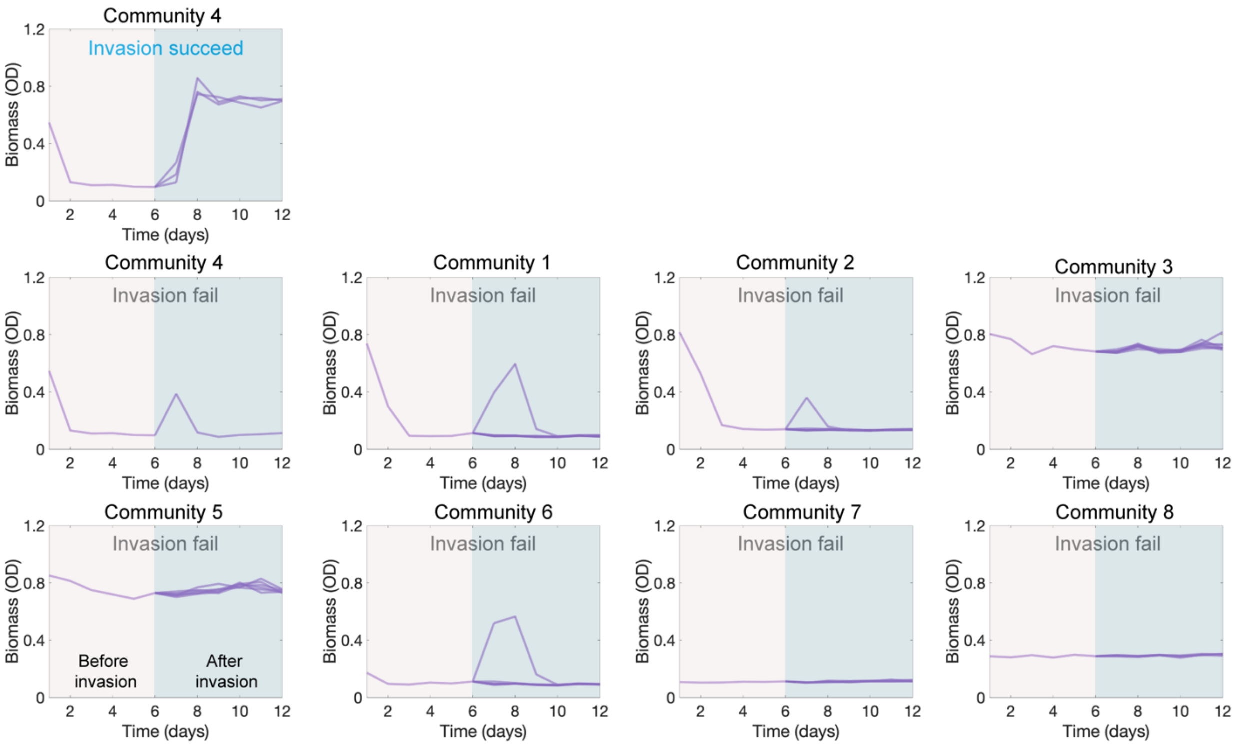

Supplementary Fig. 5. Time series for the biomass of the stable communities with species pool size *S*=12 under strong average interaction strength (high nutrients concentration). Each panel shows the time series for the OD (600nm) of one stable community with species pool size *S*=12 under high nutrient. The invaders were introduced on day 6, and the time series of successful invasions and failed invasions for the same communities were displayed in different panels.

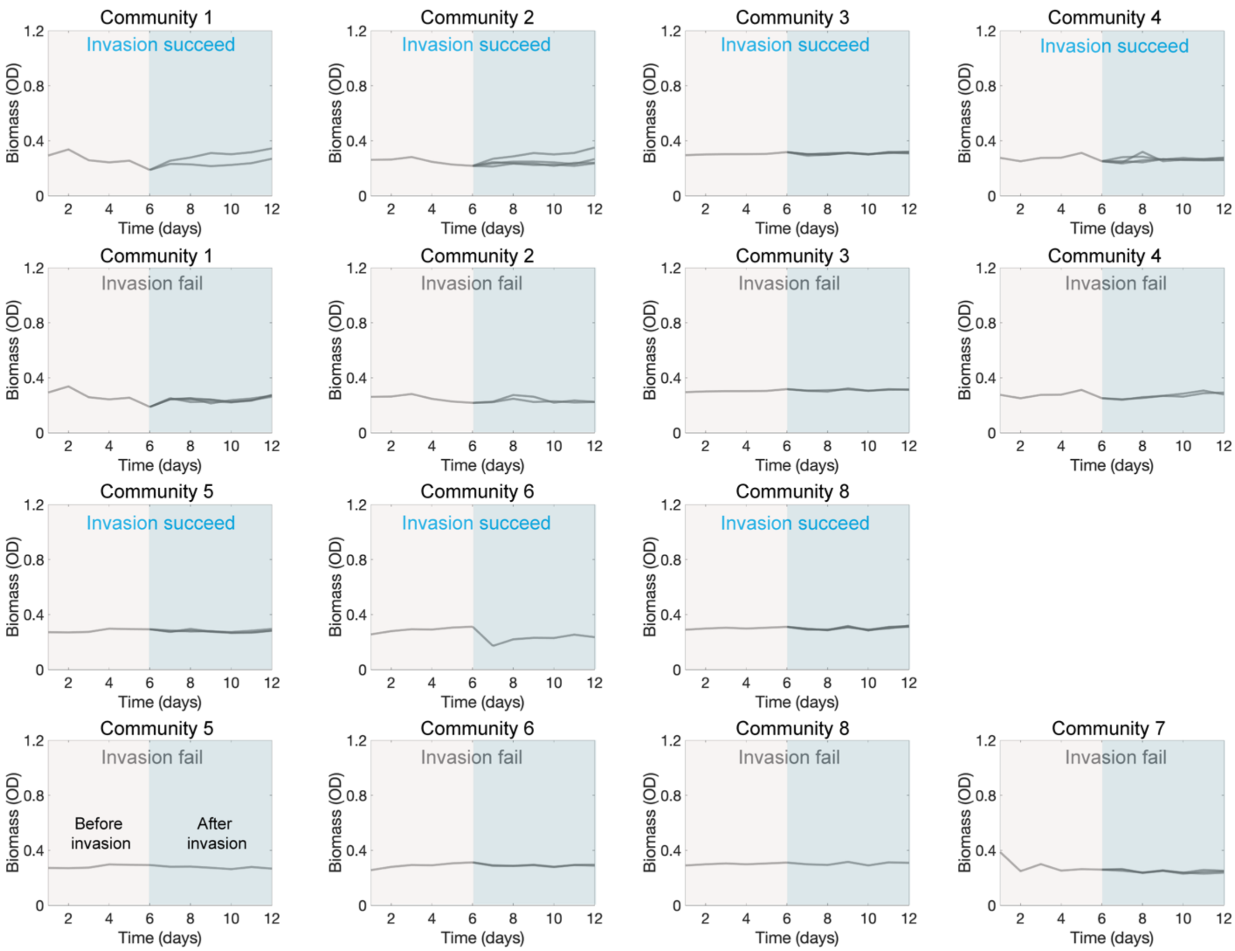

Supplementary Fig. 6. Time series for the biomass of the stable communities with species pool size *S*=20 under weak average interaction strength (low nutrients concentration). Each panel shows the time series for the OD (600nm) of one stable community with species pool size *S*=20 under low nutrient. The invaders were introduced on day 6, and the time series of successful invasions and failed invasions for the same communities were displayed in different panels.

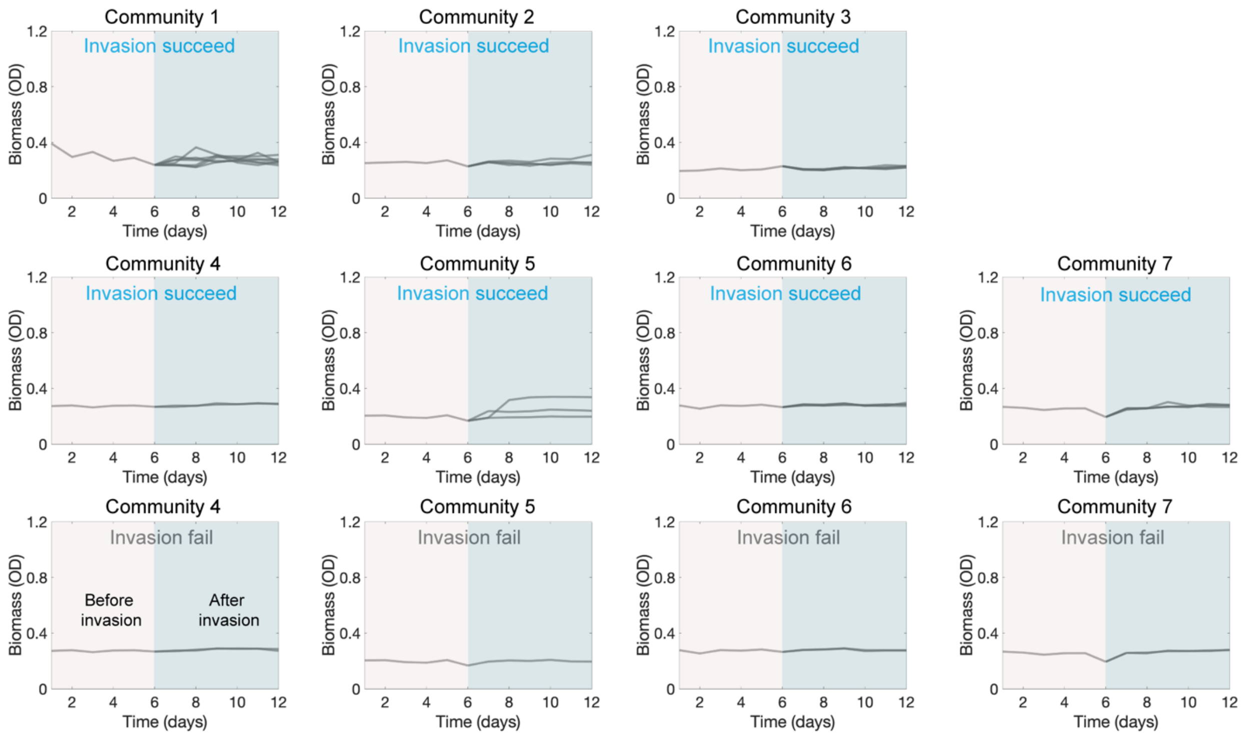

Supplementary Fig. 7. Time series for the biomass of the stable communities with species pool size *S*=12 under weak average interaction strength (low nutrients concentration). Each panel shows the time series for the OD (600nm) of one stable community with species pool size *S*=12 under low nutrient. The invaders were introduced on day 6, and the time series of successful invasions and failed invasions for the same communities were displayed in different panels.

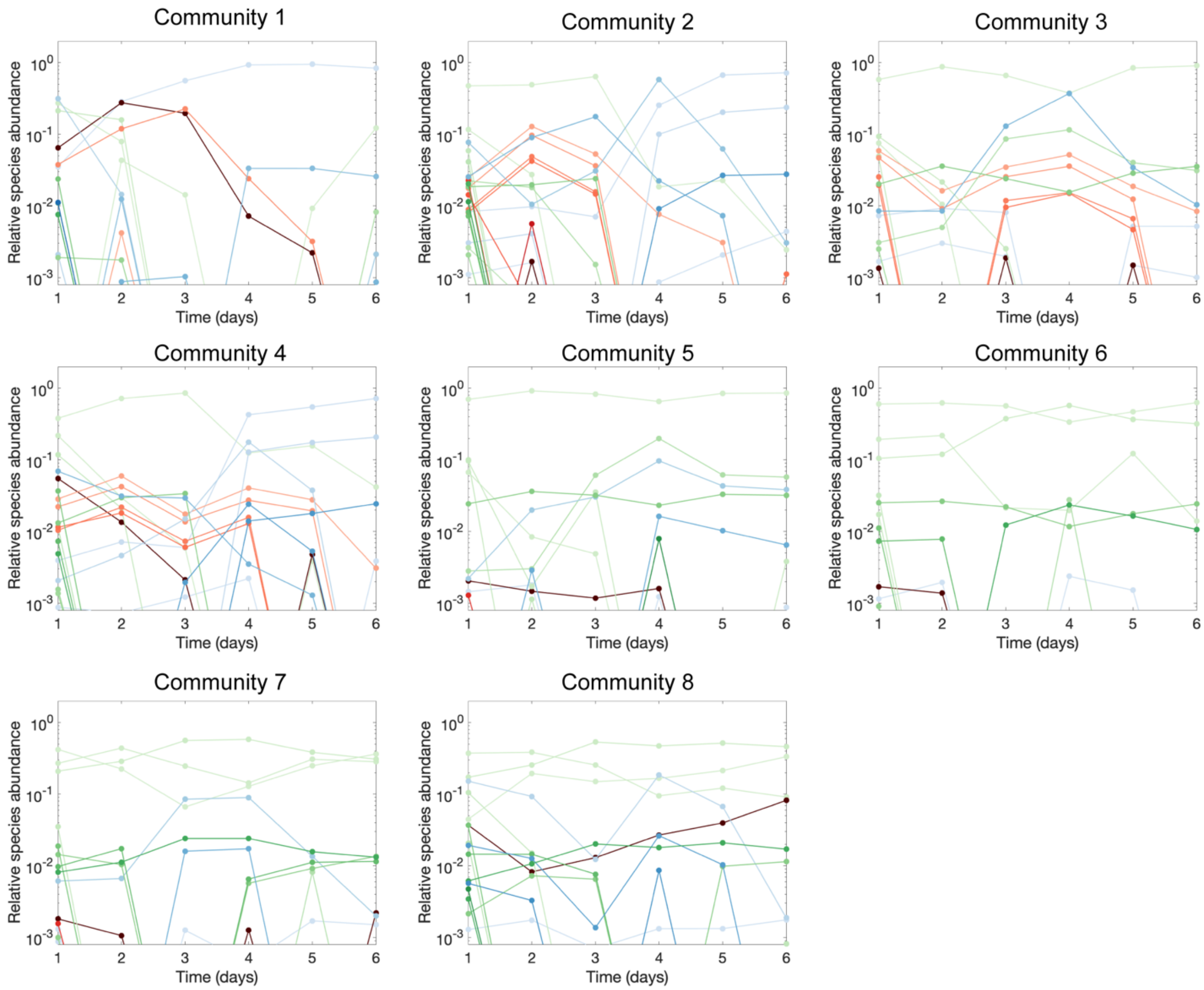

Supplementary Fig. 8. Time series for the relative species abundances of the fluctuating communities with species pool size *S*=20 under strong average interaction strength (high nutrients concentration). Each panel shows the time series for the relative species abundances of one fluctuating community before introducing invaders, where species pool size *S*=20 under high nutrient.

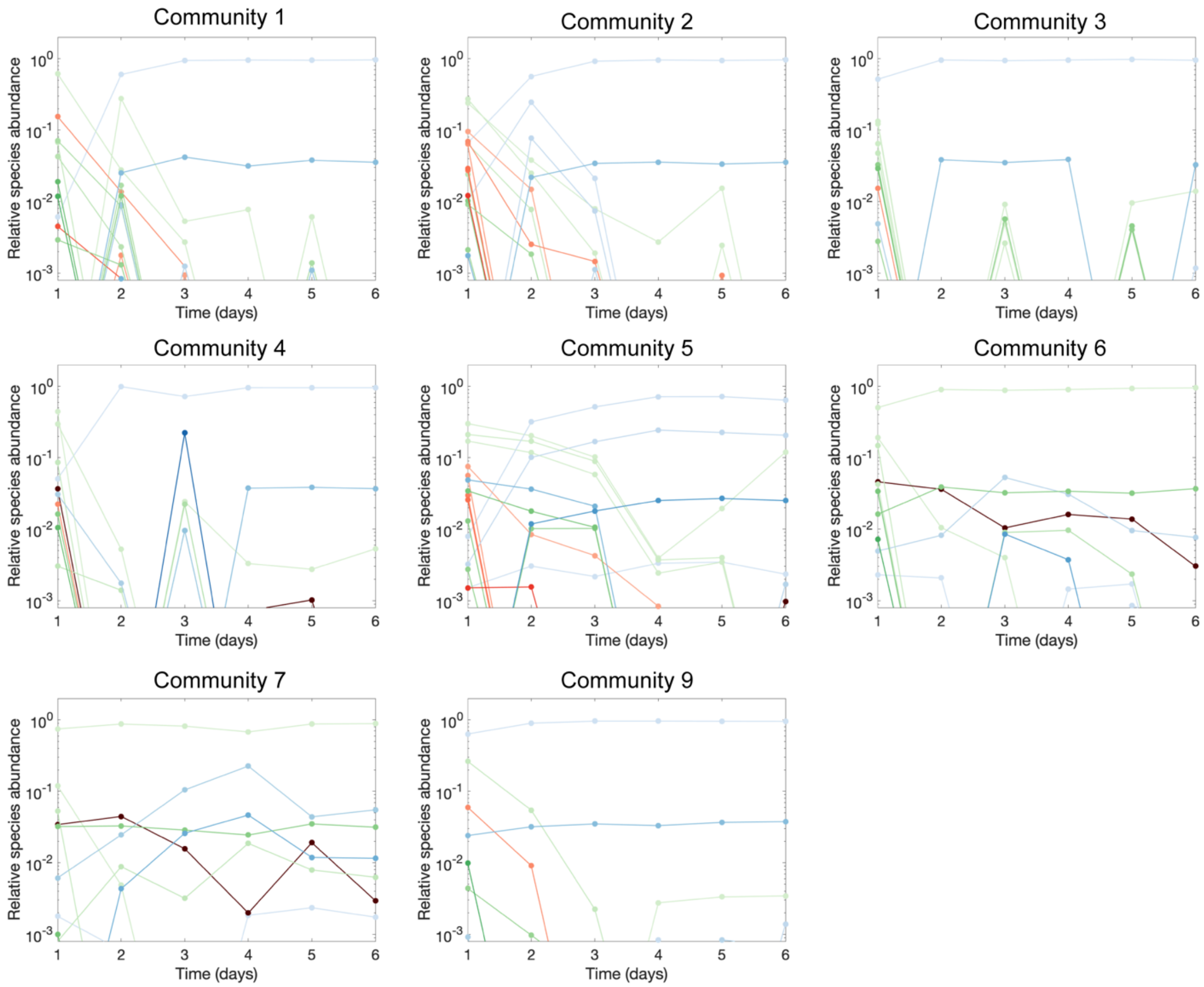

Supplementary Fig. 9. Time series for the relative species abundances of the stable communities with species pool size *S*=20 under strong average interaction strength (high nutrients concentration). Each panel shows the time series for the relative species abundances of one stable community before introducing invaders, where species pool size *S*=20 under high nutrient.

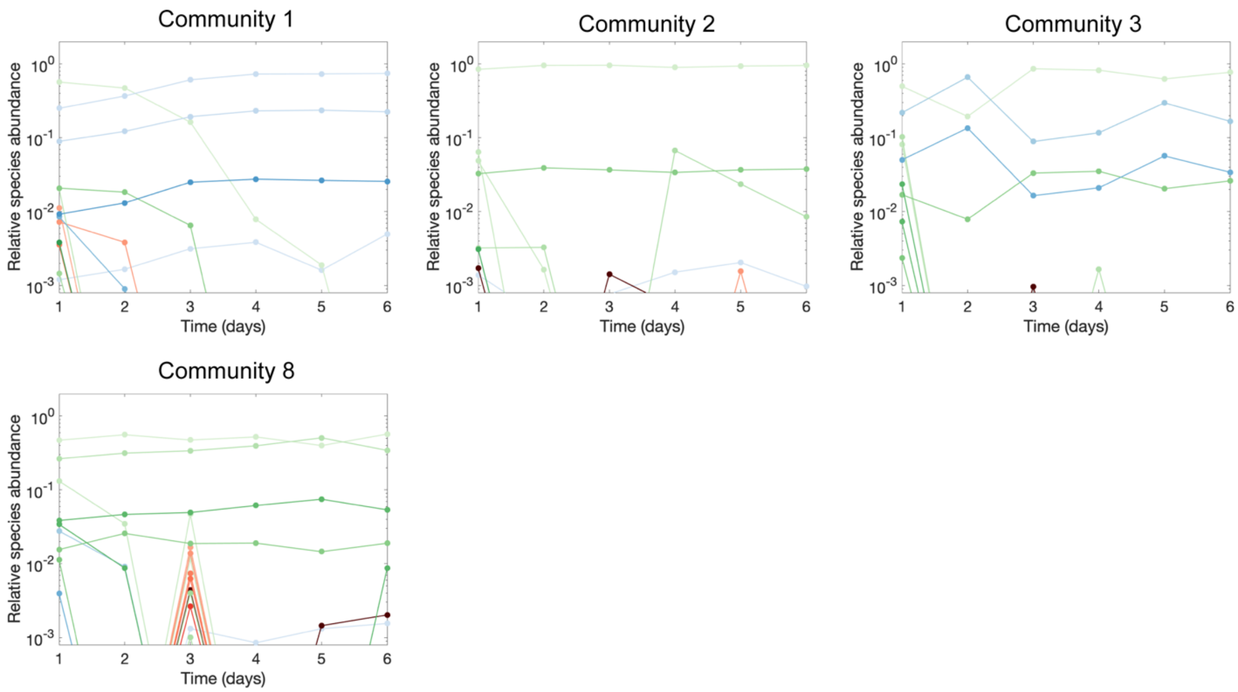

Supplementary Fig. 10. Time series for the relative species abundances of the stable communities with species pool size *S*=12 under strong average interaction strength (high nutrients concentration). Each panel shows the time series for the relative species abundances of one stable community before introducing invaders, where species pool size *S*=12 under high nutrient.

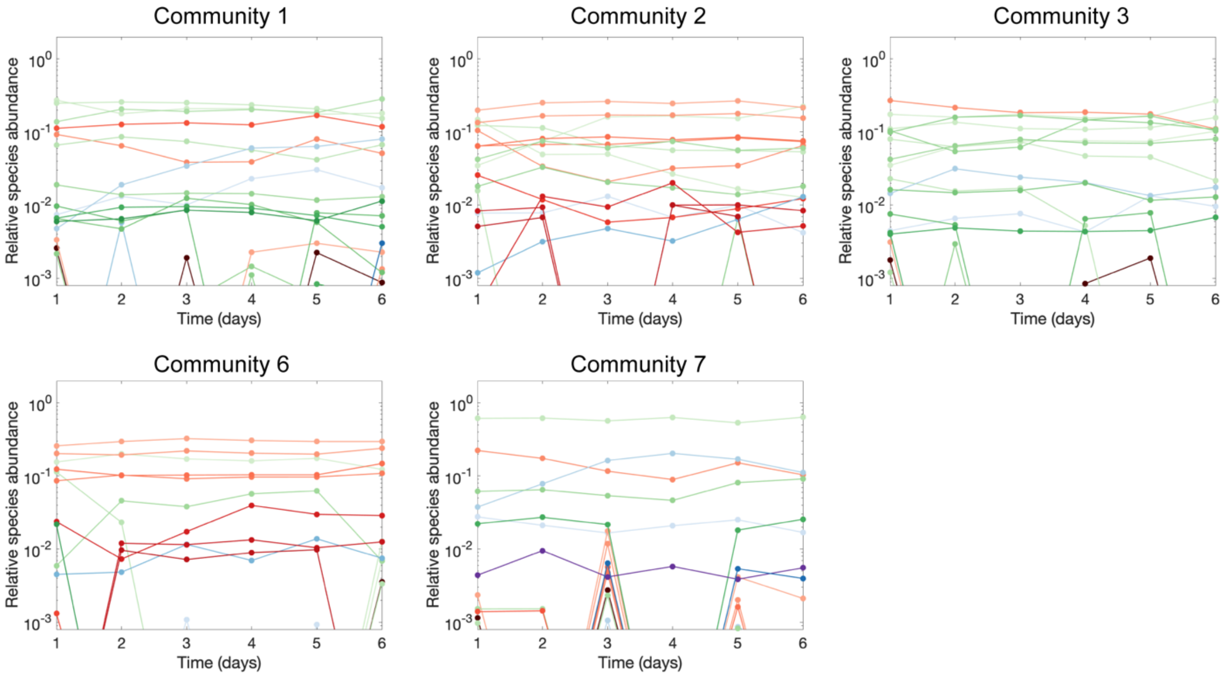

Supplementary Fig. 11. Time series for the relative species abundances of the stable communities with species pool size *S*=20 under weak average interaction strength (low nutrients concentration). Each panel shows the time series for the relative species abundances of one community before introducing invaders, where species pool size *S*=20 under low nutrient.

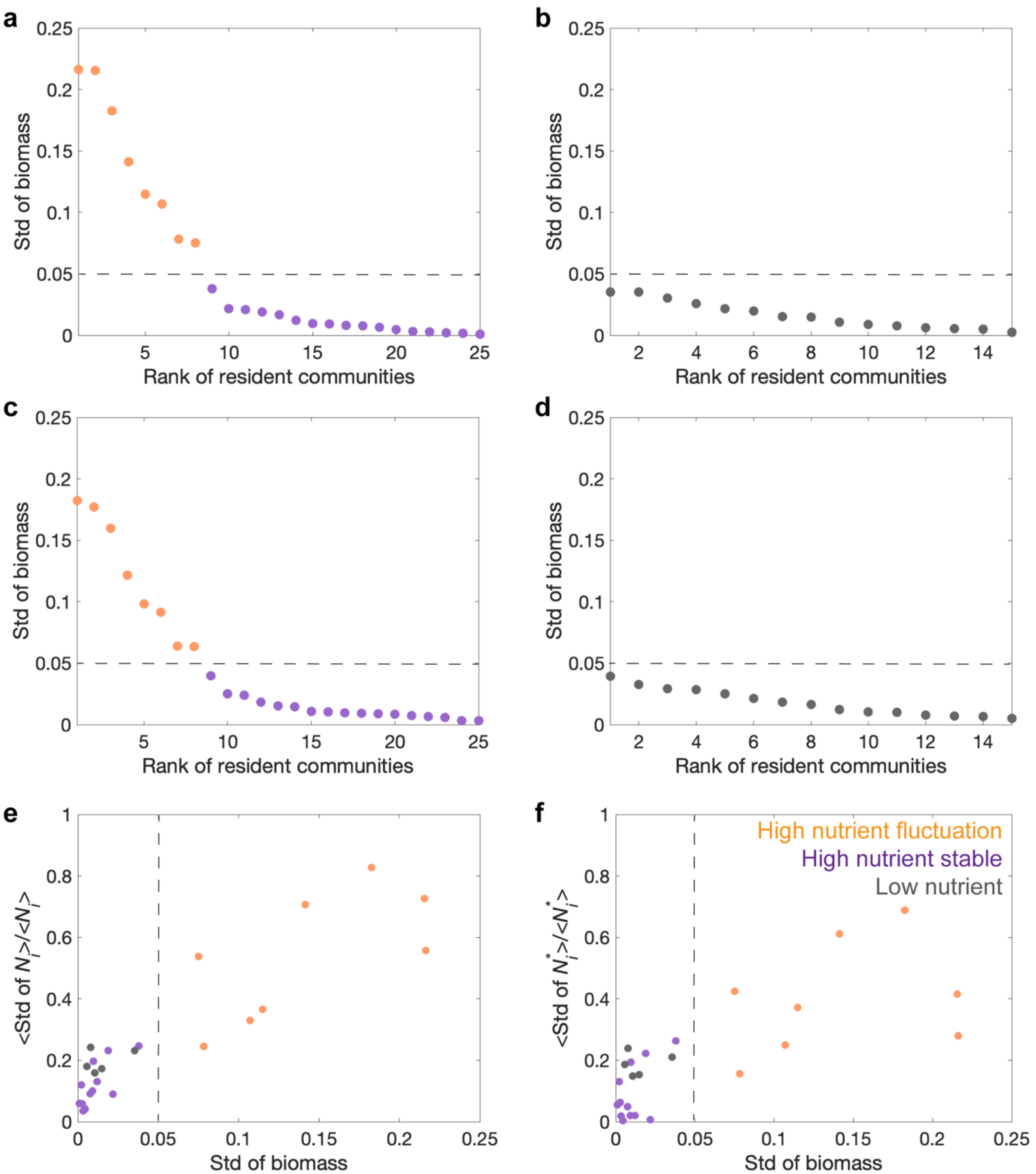

Supplementary Fig. 12. Classification of fluctuating and stable resident communities in experiment. a, The standard deviation of community biomass over day 4, day 5 and day 6 show that the stability threshold of 0.05 can separate the communities into stable ones (purple points) with small biomass deviation and fluctuating ones (orange points) with relatively large biomass deviation under high nutrient. **b**, The standard deviation of community biomass under low nutrient are small (all below the stability threshold of 0.05), which were naturally classified into stable communities. **c**, Similarly, the standard deviation of community biomass over days 3, 4, 5, and 6 under high nutrient conditions confirms that the stability threshold of 0.05 can distinguish between

stable communities (purple points) with low biomass deviation and fluctuating communities (orange points) with higher biomass deviation. **d**, Under low nutrient conditions, the standard deviation of community biomass over days 3, 4, 5, and 6 remains below the stability threshold of 0.05, consistently classifying the communities as stable. **e**, The average coefficient of (temporal) variation for absolute species abundances (N_i, computed as the product of total biomass and species relative abundance) exhibit a strong positive correlation with standard deviation of biomass in the experimental communities (correlation=0.91, p=4.67×10^{-10}). *K*-means clustering method classifies the points into two clusters where fluctuating communities locate on top right region and stable communities locate on bottom left region. **f**, The average coefficient of (temporal) variation for relative species abundances (N_i^*, relative species abundance through 16s sequencing) also exhibits a strong positive correlation with standard deviation of biomass in the experimental communities (correlation=0.76, p=1.06×10^{-5}). *K*-means clustering method classifies the communities into stable ones (purple and gray points) and fluctuating ones (orange points). The results suggest that fluctuation in community biomass cooccurs with fluctuation in relative species abundances.

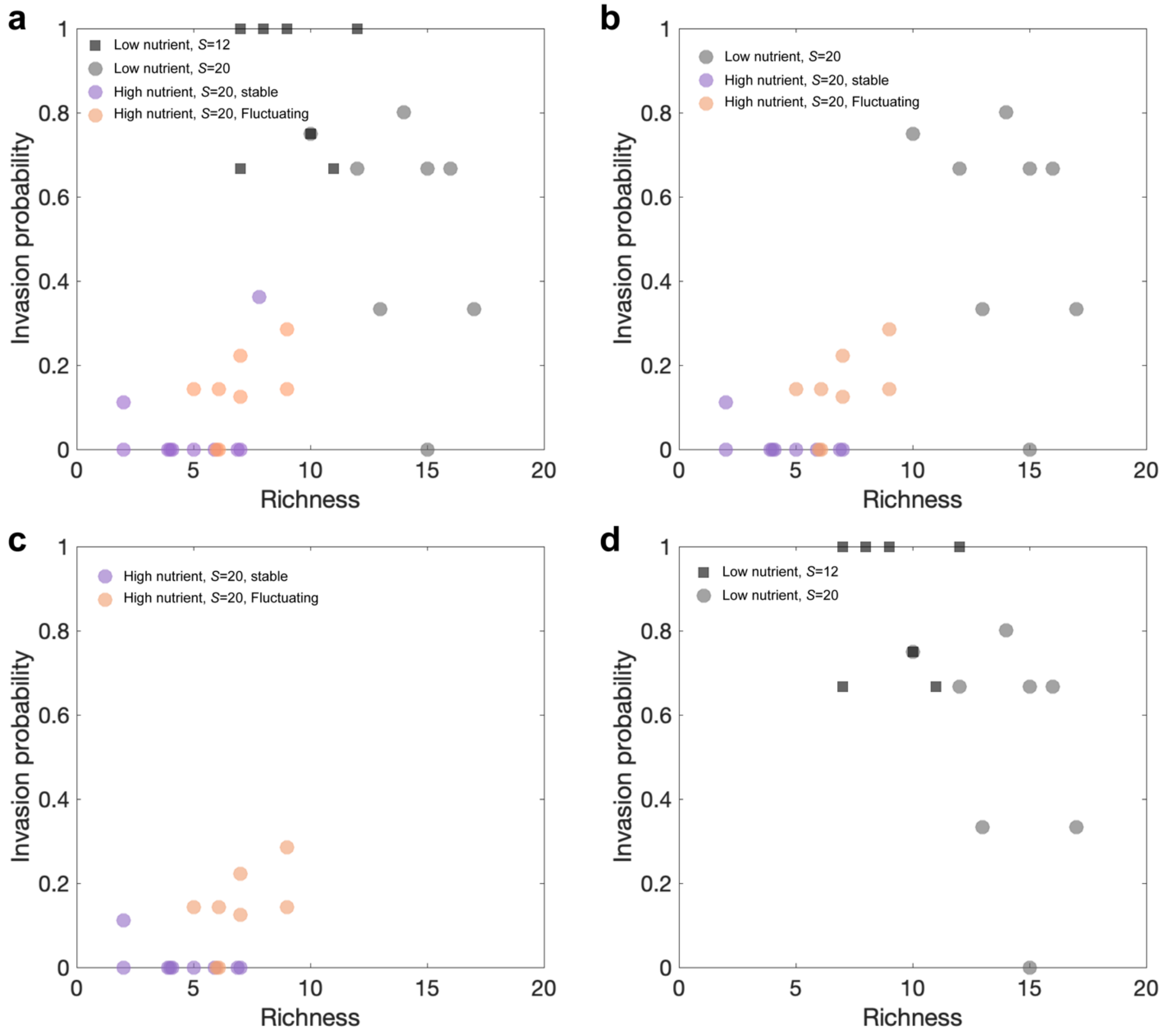

Supplementary Fig. 13. Different invasibility-richness relationships in experiment depending upon how the richness is changed (a). b, Invasibility positively correlates with richness when varying interaction strength under fixed species pool (positive correlation between *S*=20 communities under low and high nutrient, correlation is calculated across all data points in panel **b**, correlation coefficient=0.7, p=1.06×10^{-4}). **c**, Invasibility positively correlates with richness when randomly sample *S*=20 communities under high nutrient, due to fluctuating communities display larger richness and larger invasion probability (correlation is calculated across all data points in panel **c**, correlation coefficient=0.5, p=0.047). Neither stable communities (purple points in panel **c**) nor fluctuating communities (orange points in panel **c**) display any statistically significant correlation between diversity and invasibility (p=0.16 for stable and p=0.15 for fluctuating). **d**, Invasibility negatively correlates with richness when increasing species pool size from *S*=12 to *S*=20 under low nutrient (correlation is calculated across all data points in panel **d**, correlation coefficient=-0.62, p=0.014). Two-sided test for statistical significance was performed.

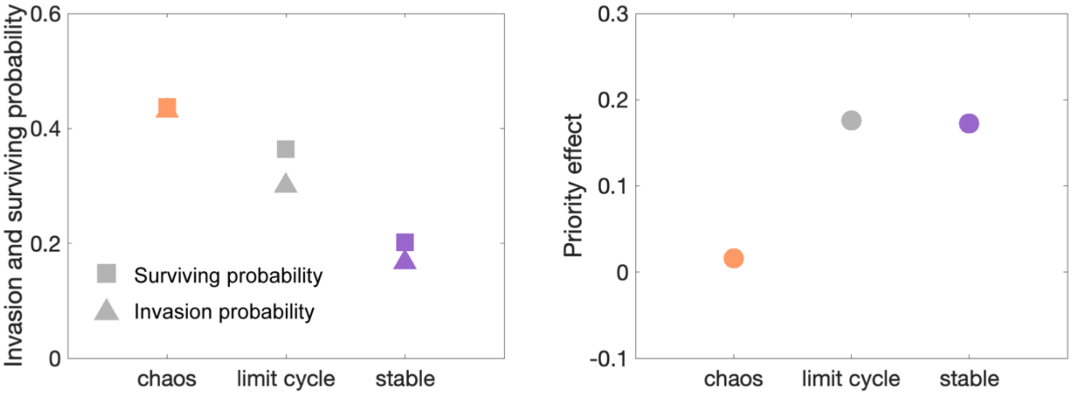

Supplementary Fig. 14. Priority effect originates from alternative stable states and limit cycle oscillations rather than chaotic fluctuations in simulations. Lotka-Volterra model simulations show that both surviving probability and invasion probability increase as community dynamics transition from alternative stable states to limit cycle oscillations and to chaos. Communities with chaotic fluctuations in species abundance do not display significant priority effect which can be explained by its ergodicity [41,43], whereas communities with limit cycle oscillations and alternative stable states both show significant priority effect. The simulation in this figure was performed under S=40 and $<\alpha_{ij}>$=0.65 over 1000 replicates, among which we observed 223 chaotic fluctuating communities, 340 limit cycle oscillations, and 437 alternative stable states. The fluctuating communities were classified into chaos when its Lyapunov exponent is positive, while classified into limit cycle when its Lyapunov exponent is negative. As studied and shown in previous theoretical studies[54,41,43], ergodicity in the context of chaotic fluctuations means that the community's state is memoryless. The dynamical trajectories of species composition do not depend on the community's history and do not reach alternative stable states or alternative dynamical attractors. Therefore, different orders of species arrival or different initial species compositions do not lead to different community states in the chaotic fluctuation regime

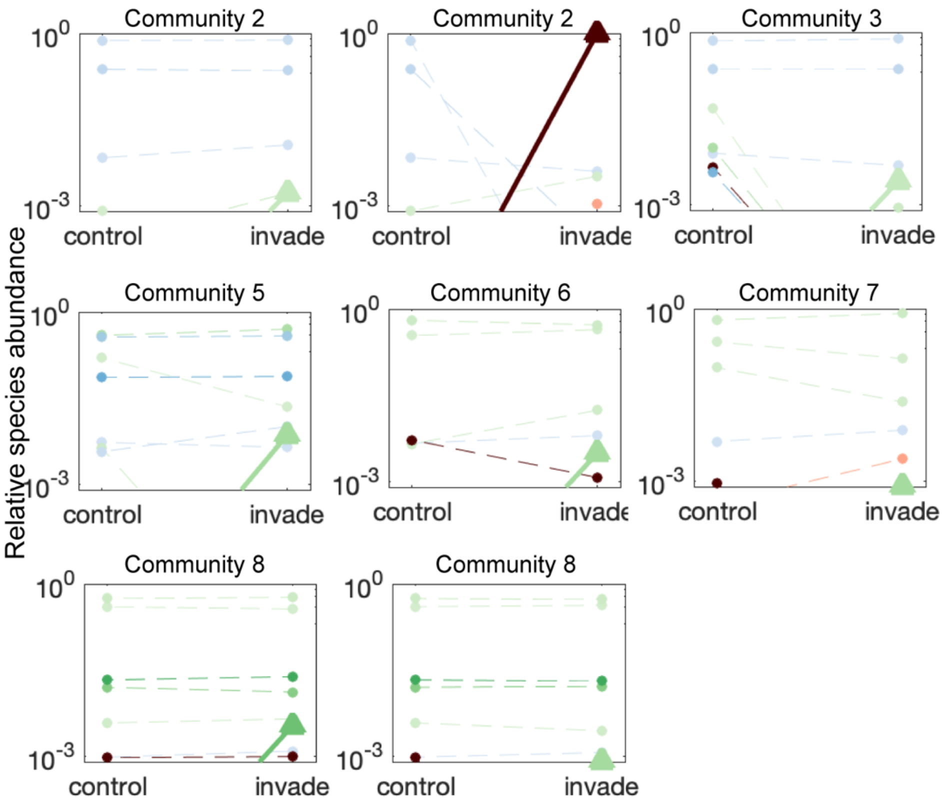

Supplementary Fig. 15. Successful invasions lead to change in species composition in fluctuating communities with *S*=20 under high nutrient, which can be shown by comparing the relative species abundance between invaded communities and control communities without introducing invader. The circles and triangles in the figure represent resident species and invader species, respectively. The successful invasions can cause the extinction of other resident species (circles drop below the extinction threshold under invasion) and the colonization of other resident species (circles go beyond the extinction threshold under invasion).

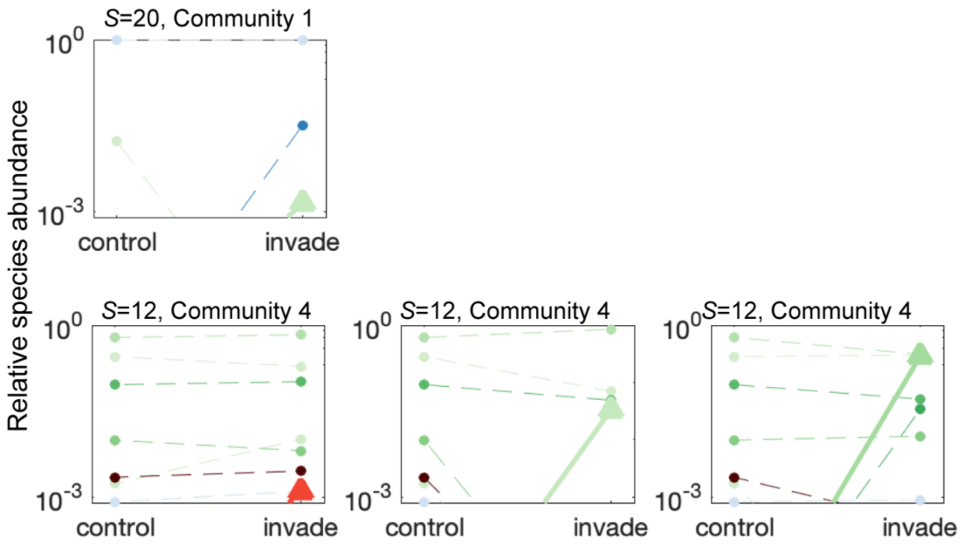

Supplementary Fig. 16. Successful invasions lead to change in species composition in stable communities under high nutrient, which can be shown by comparing the relative species abundance between invaded communities and control communities without introducing invader. The circles and triangles in the figure represent resident species and invader species, respectively. The successful invasions can cause the extinction of other resident species (circles drop below the extinction threshold under invasion) and the colonization of other resident species (circles go beyond the extinction threshold under invasion).

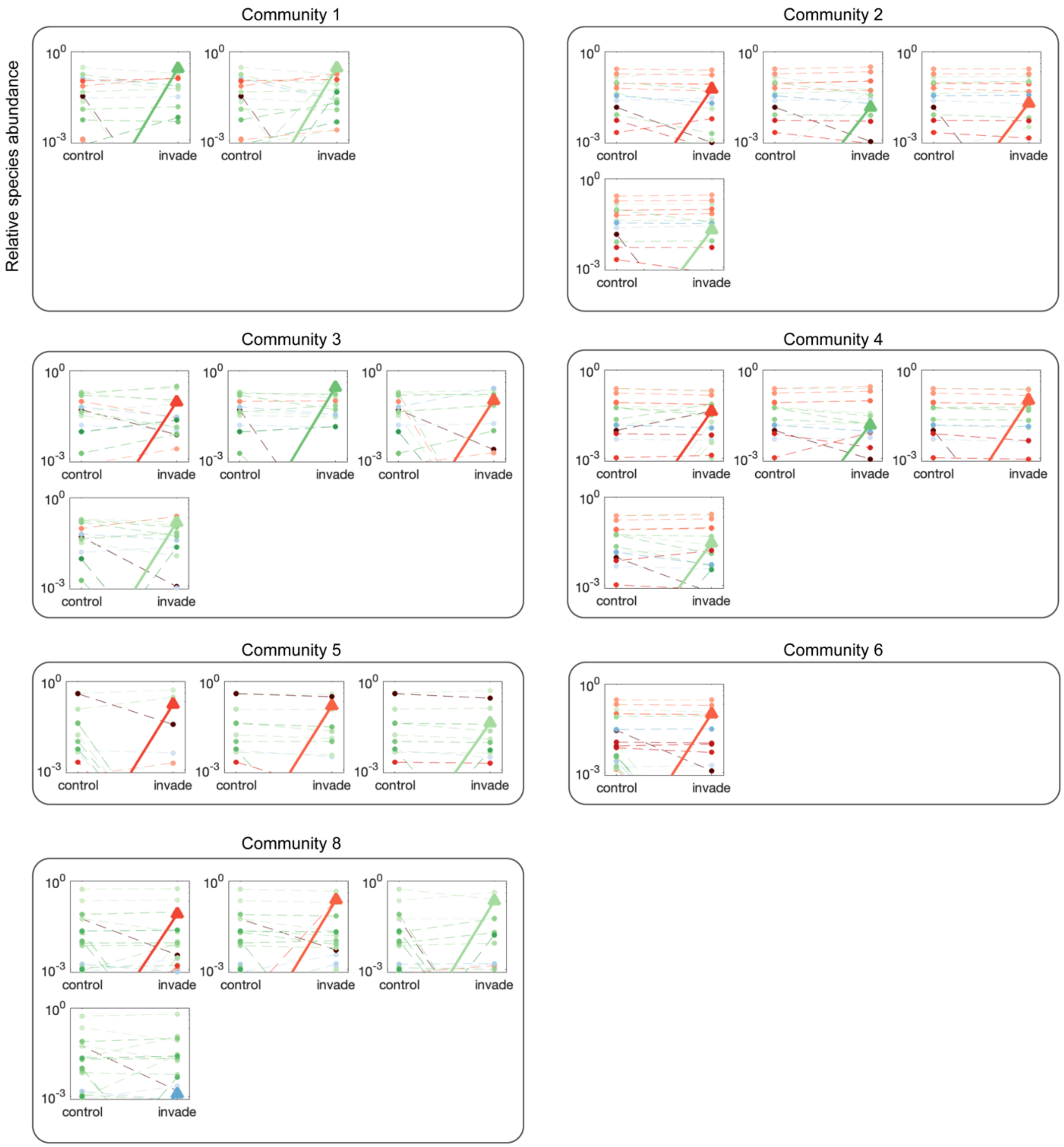

Supplementary Fig. 17. Successful invasions lead to change in species composition in communities with *S*=20 under low nutrient, which can be shown by comparing the relative species abundance between invaded communities and control communities without introducing invader. The circles and triangles in the figure represent resident species and invader species, respectively. The successful invasions can cause the extinction of other resident species (circles drop below the extinction threshold under invasion) and the colonization of other resident species (circles go beyond the extinction threshold under invasion).

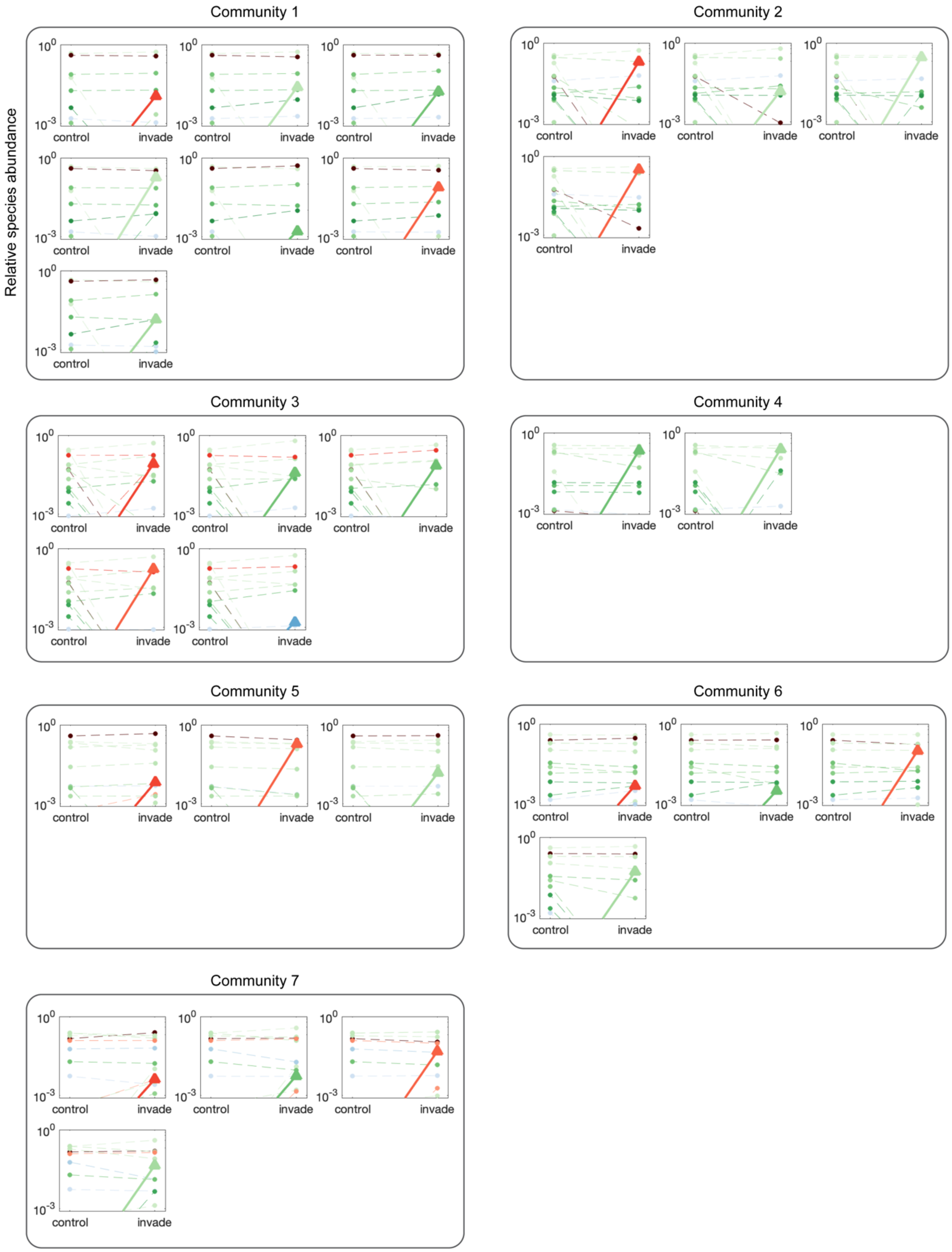

Supplementary Fig. 18. Successful invasions lead to change in species composition in communities with *S*=12 under low nutrient, which can be shown by comparing the relative

species abundance between invaded communities and control communities without introducing invader. The circles and triangles in the figure represent resident species and invader species, respectively. The successful invasions can cause the extinction of other resident species (circles drop below the extinction threshold under invasion) and the colonization of other resident species (circles go beyond the extinction threshold under invasion).

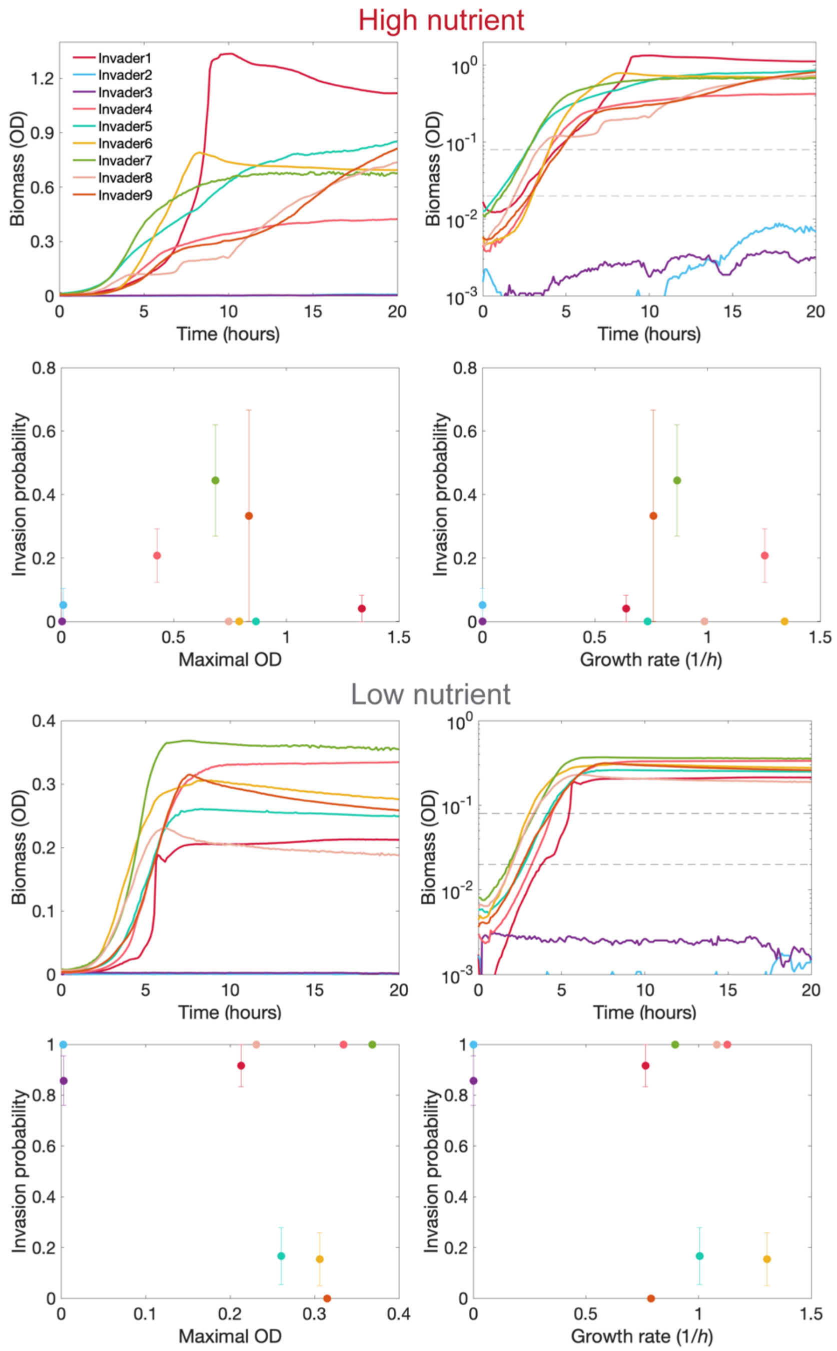

Supplementary Fig. 19. There is no correlation between the invasiveness of invaders and their carrying capacities and growth rates. The growth curves of invaders were measured after a dilution of 10^5 folds. The carrying capacity of invaders were quantified by the maximal OD over 24 hours of growth. The growth rates of invaders were quantified by fitting the slopes of growth curves between the two horizontal dashed lines in the figure on logarithmic scale for biomass. There is no statistically significant correlation between invasion probability of invaders with their carrying capacities and growth rates, under both high nutrient and low nutrient. The correlation coefficient between invasion probability and invader growth rates is 0.212, p-value=0.584 under high nutrient; correlation coefficient=-0.334, p-value=0.380 under low nutrient. The correlation coefficient between invasion probability and invader carrying capacities is 0.076, p-value=0.846

under high nutrient; correlation coefficient=-0.324, p-value=0.394 under low nutrient. The phylogeny of invader 1 to invader 9 are: Flectobacillus, Pseudomonas, Pedobacter, Pseudomonas, Pantoea, Bacillus, Enterobacterales, Pantoea, Chryseobacterium. To see the Allee effect for invaders 2 and 3, notice that the growth curves of invaders 2 and 3 (blue and purple curves) indicate that they do not grow to a well-detectable signal on the OD reader over 20 hours, demonstrating an Allee effect of these two species. Two-sided test for statistical significance was performed.

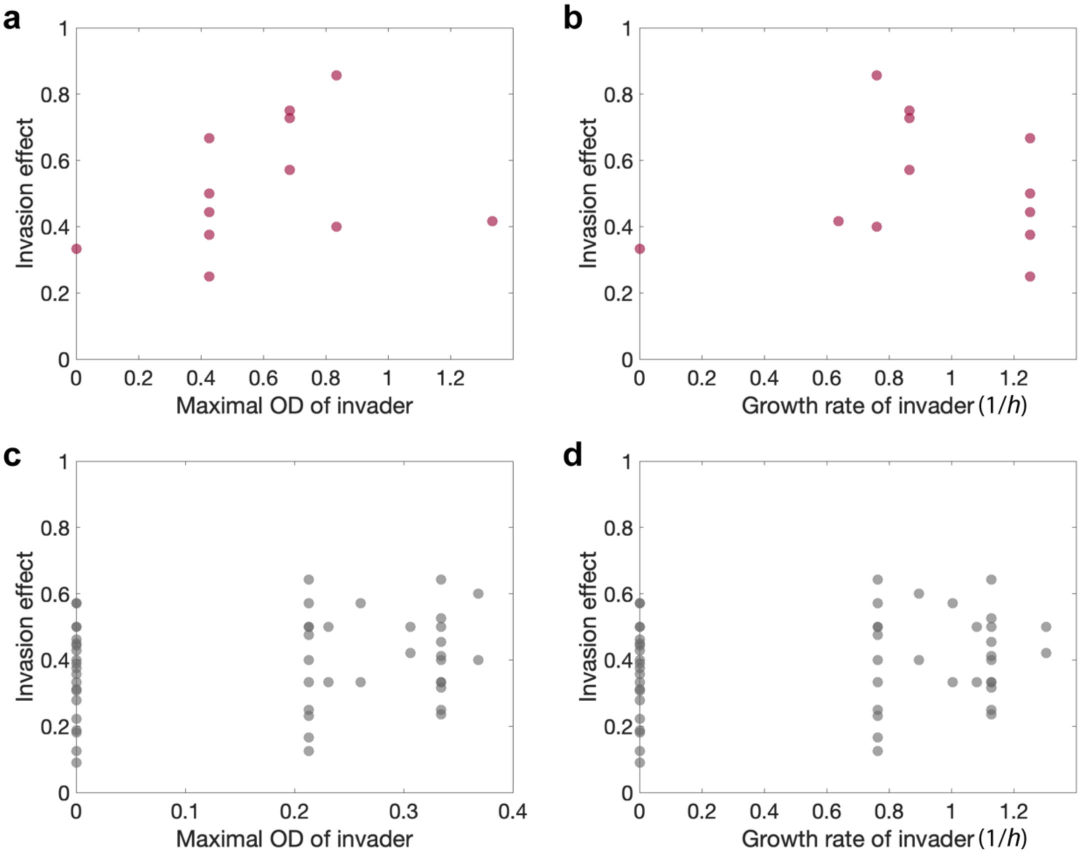

Supplementary Fig. 20. There is no statistically significant correlation between invasion effect and invader properties. Under high nutrient, invasion effect does not show statistically significant correlation with carrying capacity (a) (correlation coefficient=0.281, p-value=0.377) and growth rate (b) (correlation coefficient=0.023, p-value=0.944). Under low nutrient, invasion effect does not show statistically significant correlation with carrying capacity (c) (correlation coefficient=0.208, p-value=0.143) and growth rate (d) (correlation coefficient=0.200, p-value=0.160).

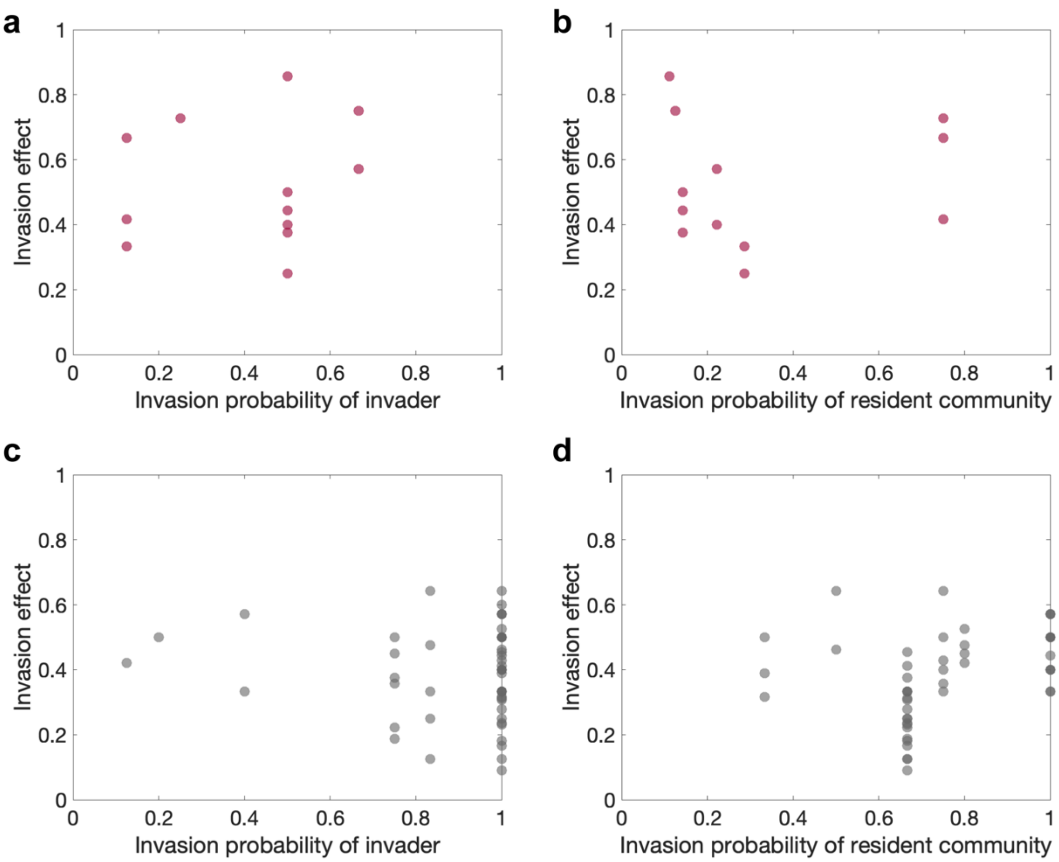

Supplementary Fig. 21. There is no statistically significant correlation between invasion effect and invasion probability. Under high nutrient, invasion effect does not show statistically significant correlation with invasion probability of invaders (a) (correlation coefficient=0.127, p-value=0.694) and invasion probability of resident communities (b) (correlation coefficient=0.105, p-value=0.745). Under low nutrient, invasion effect does not show statistically significant correlation with invasion probability of invaders (c) (correlation coefficient=-0.086, p-value=0.550) and invasion probability of resident communities (d) (correlation coefficient=318, p-value=276). Two-sided test for statistical significance was performed.

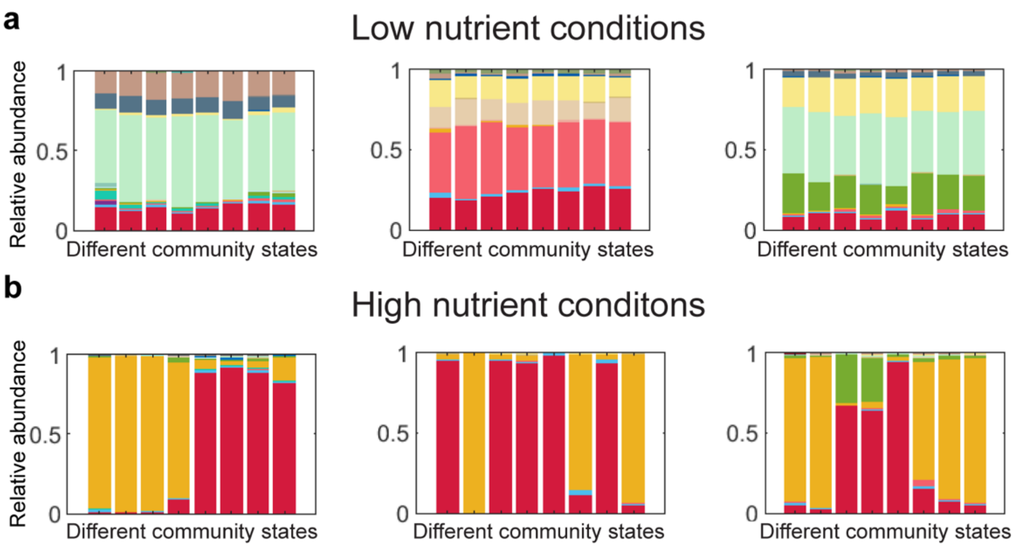

Supplementary Fig. 22. Communities reach global stable states under low nutrient conditions (weak interaction) but reach alternative stable states under high nutrient conditions (strong interaction) when starting from different initial species compositions. Each bar represents a final community stable state after 7 daily dilution cycles starting from a particular initial condition. The communities assemble from the same species in the original pool (*S*=12 for the left and middle communities, *S*=24 for the right communities), under low-nutrient (weak interaction) and high-nutrient (strong interaction) conditions, respectively. For each initial condition, one of the species occupies 99% volume of the initial inoculum, while the other species in the pool together occupy only 1% volume of the initial inoculum. The results show that these very different initial species compositions lead to the same global stable state under low nutrient conditions (weak interaction) but result in alternative stable states under high nutrient conditions (strong interaction). This indicates the presence of alternative stable states and priority effect under high nutrient (strong interaction), where the initial composition influences the final community structure.

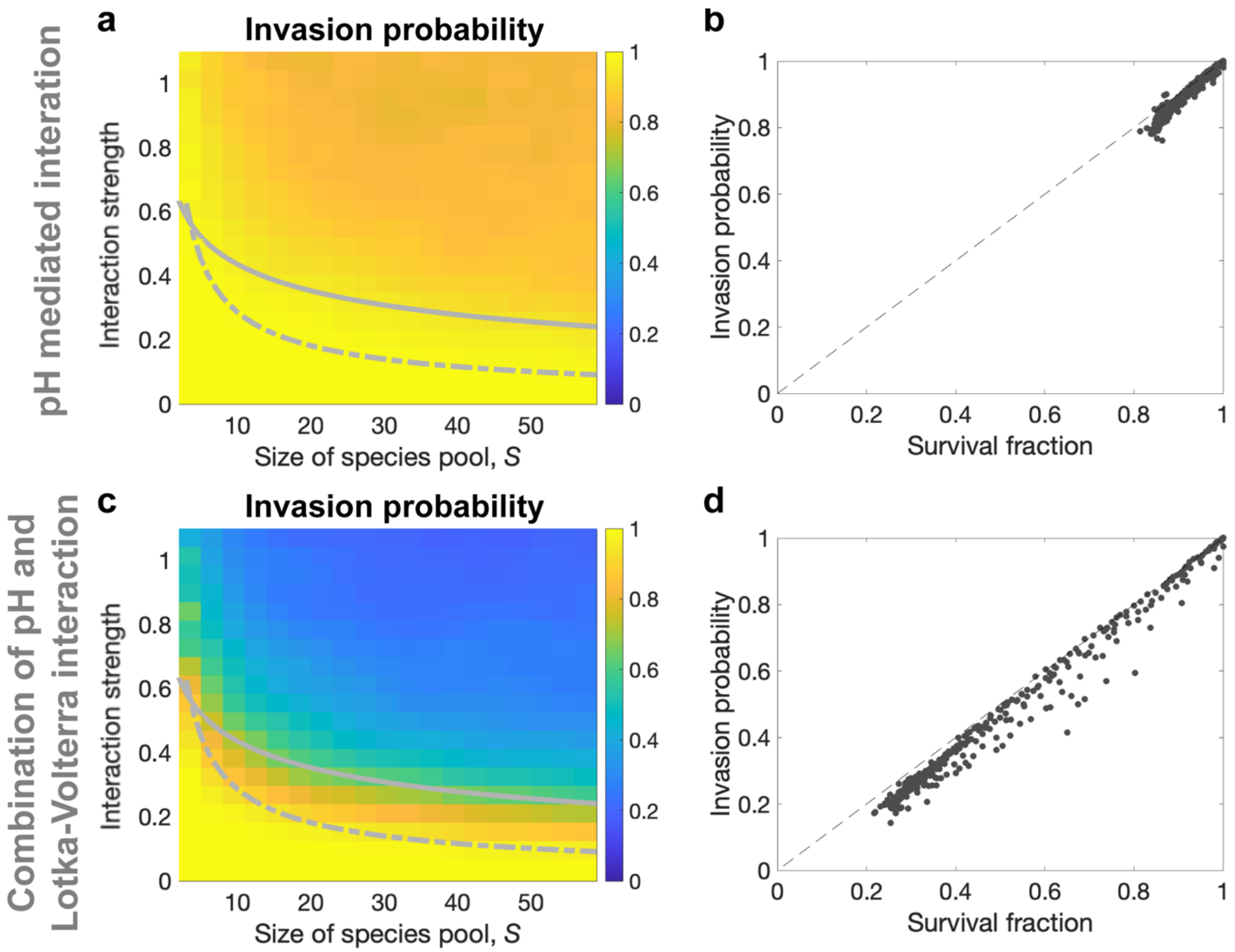

Supplementary Fig. 23. The invasion probability decreases with increasing pH-mediated interaction strength and gLV-type interaction strength, while the survival fraction remains approximately equal to the invasion probability. **a**, The pure pH model, without gLV-type interactions, predicts that invasion probability decreases with increasing pH interaction strength (mean of $\beta^* g_i k_i$) and species pool size. **b**, In the pure pH model, the survival fraction is approximately equal to the invasion probability, even without gLV-type interactions. **c**, When both pH interaction strength (mean of $\beta^* g_i k_i$) and gLV-type interaction strength (mean of α_{ij}) increase simultaneously, the invasion probability decreases. **d**, The survival fraction remains approximately equal to the invasion probability when combining the pH model with the gLV model. The points and color maps depict the mean value over 100 simulations.

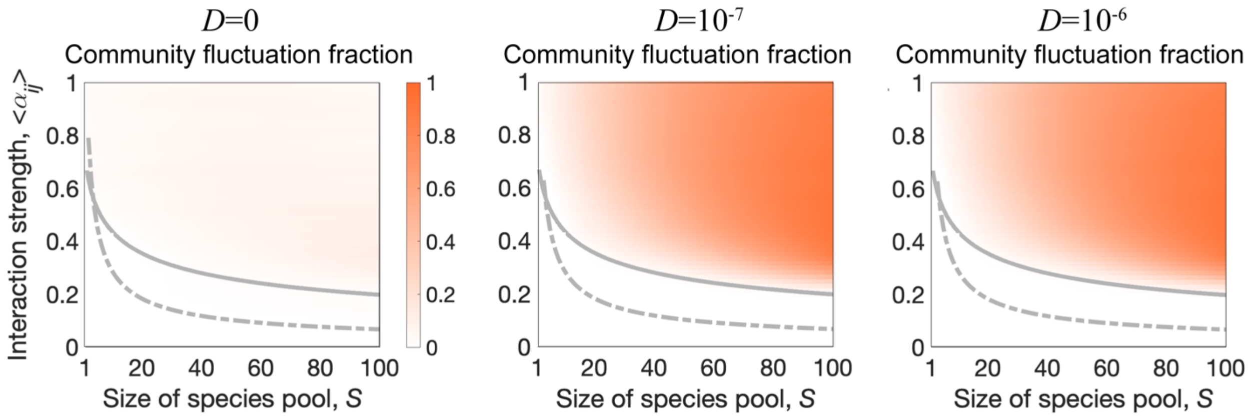

Supplementary Fig. 24. Non-zero dispersal sustains persistent community fluctuations in gLV model. The panels show the theoretical phase diagrams of community fluctuation fraction under different dispersal rates (D=0, D=10^{-7}, D=10^{-6}). Communities under no dispersal (D=0, left panels) exhibit a low fluctuation fraction in the persistent fluctuation phase. The patterns of ecological diversity and dynamics do not significantly change as the dispersal rate varies from D=10^{-7} (middle panels) to D=10^{-6} (right panels). The dashed line and solid line in the figures represent survival boundary and stability boundary, respectively. The color maps depict the mean value over 1000 simulations.

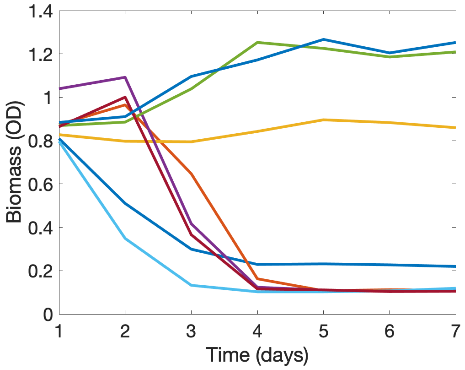

Supplementary Fig. 25. Zero dispersal leads to stable communities under high nutrient conditions (strong interactions) in the experiment. The biomass of eight distinct microbial communities (S=24) consistently reaches stable states when exposed to high nutrient concentrations (strong interactions) in the absence of species dispersal from the species pool to the community. In contrast, when dispersal is present, a significant proportion of communities exhibit fluctuations under the same high nutrient conditions and species pool size, as previously reported in Fig. 1d and in our previous paper [40] (Fig. 2C).

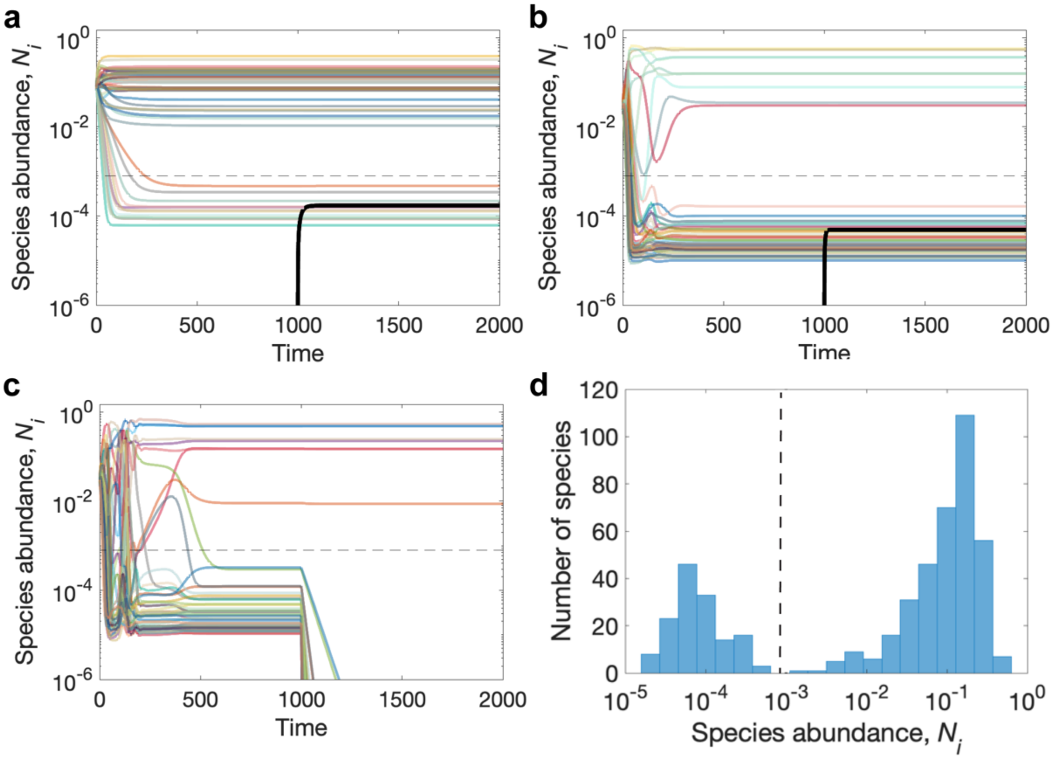

Supplementary Fig. 26. The survival threshold efficiently separates surviving species from extinct species, where the species abundances display a bimodal distribution in the simulations. a, The extinction threshold of 8×10^{-4} (horizontal dashed line) clearly separates the high-abundant, surviving species from the low-abundant "extinct" species (S=50, $<\alpha_{ij}>$=0.2). Such "extinct" species would reach zero abundance if dispersal is interrupted (**c**). **b**, The extinction threshold of 8×10^{-4} (horizontal dashed line) similarly separates the high-abundant, surviving species from the low-abundant "extinct" species under different interaction strength (S=50, $<\alpha_{ij}>$=0.6). **c**, After stopping dispersal at t=1000, only species above the extinction threshold survive with stable abundances, while the others undergo extinction (S=50, $<\alpha_{ij}>$=0.6). This demonstrates that the extinction threshold of 8×10^{-4} efficiently classifies surviving species versus those that would go extinct without dispersal. **d**, The histogram shows the number of species exhibiting the indicated abundances at steady state. The dataset was generated from 10 *in silico* communities randomly sampled (S=50, $<\alpha_{ij}>$=0.2).

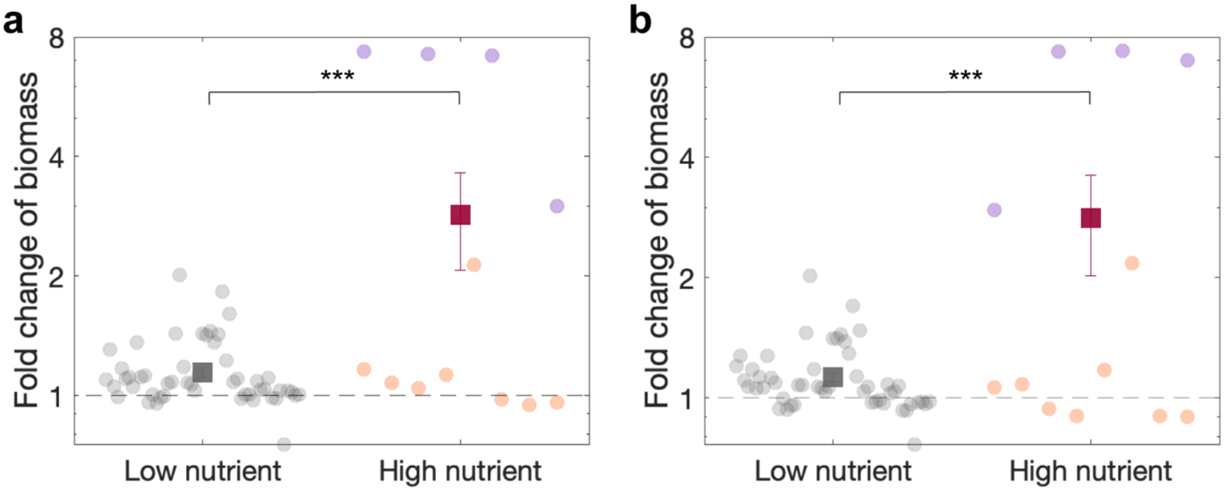

Supplementary Fig. 27. The mean fold change in biomass under high nutrient conditions is greater than under low nutrient conditions, which is robust to the choice of time points. a, Invasions into resident communities under low nutrient conditions (weak interactions) result in a statistically lower fold change in biomass compared to communities under high nutrient conditions (strong interactions) ($p = 2.9\times10^{-5}$). The number of successful invasions is n=51 (low nutrient) and n=11 (high nutrient). The fold change in biomass was calculated by comparing the biomass on day 6 (before invasion) with that on day 12 (after invasion colonization was fully established) across all communities. **b**, Similarly, invasions into resident communities under low nutrient conditions (weak interactions) cause a statistically lower fold change in biomass than those under high nutrient conditions (strong interactions) ($p = 3.5\times10^{-5}$). The fold change was calculated by comparing the biomass on day 6 (before invasion) with the average biomass of days 10, 11, and 12 (after invader colonization) across all communities. Error bars represent s.e.m.. Two-sided Student's T test was performed.

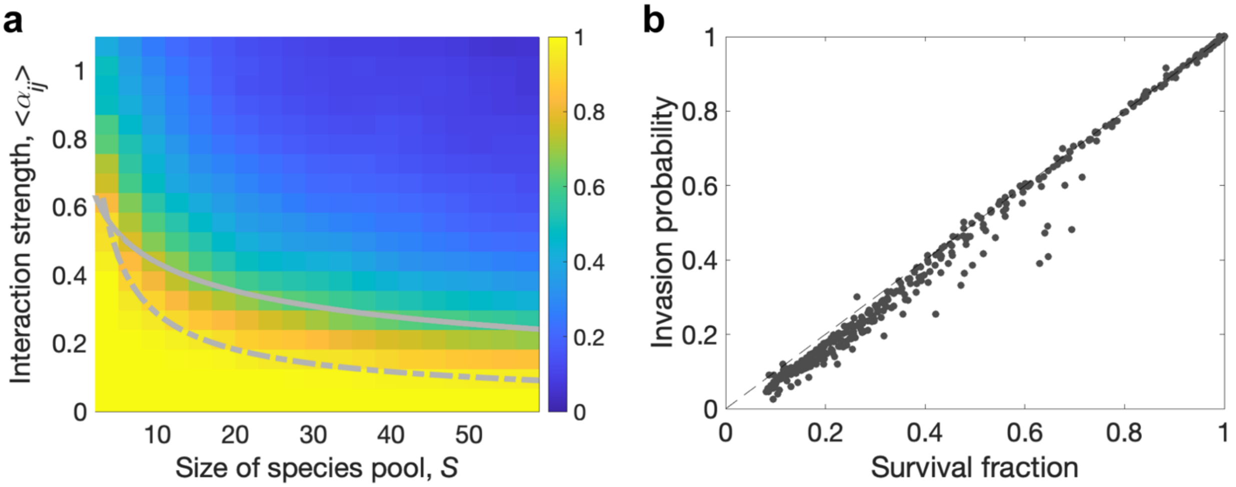

Supplementary Fig. 28. Under serial dilutions in the gLV model, the invasion probability decreases with increasing interaction strength and species pool size, the survival fraction remains approximately equal to the invasion probability. a, Invasion probability decreases with increasing interaction strength and species pool size under serial dilutions. **b**, The survival fraction is approximately equal to the invasion probability under serial dilutions in the gLV model. The points and color maps depict the mean value over 100 simulations.

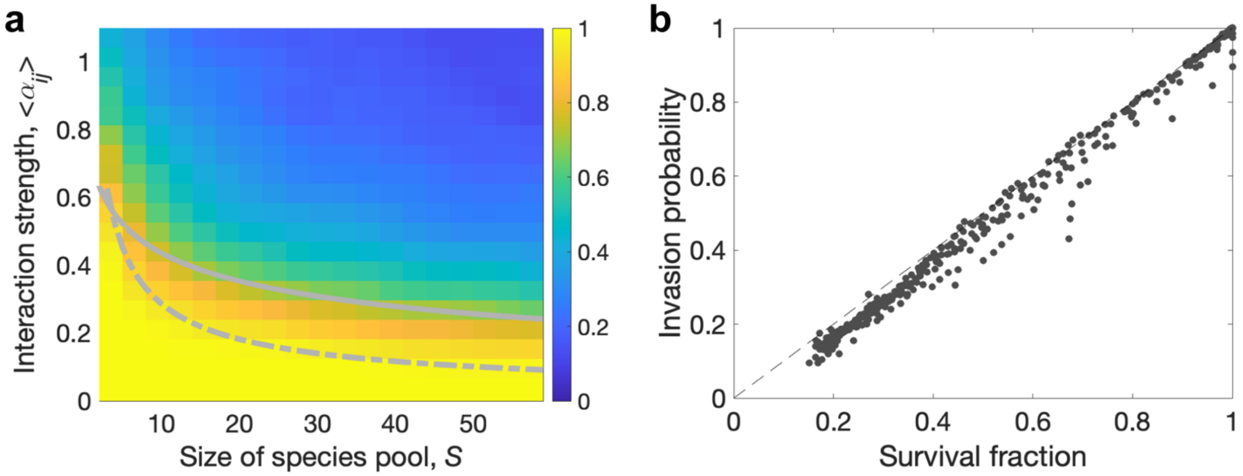

Supplementary Fig. 29. The invasion probability decreases with increasing interaction strength and species pool size across different time windows, with the survival fraction remaining approximately equal to the invasion probability. a, The invasion probability decreases as interaction strength and species pool size increase. b, The survival fraction closely mirrors the invasion probability. To assess whether invader or resident species survived, we identified species whose abundance exceeded the extinction threshold at any point during the last 24 time units of the simulation. This approach yielded invasion probability patterns consistent with those observed in a 100-unit time window. The points and color maps depict the mean value over 100 simulations.

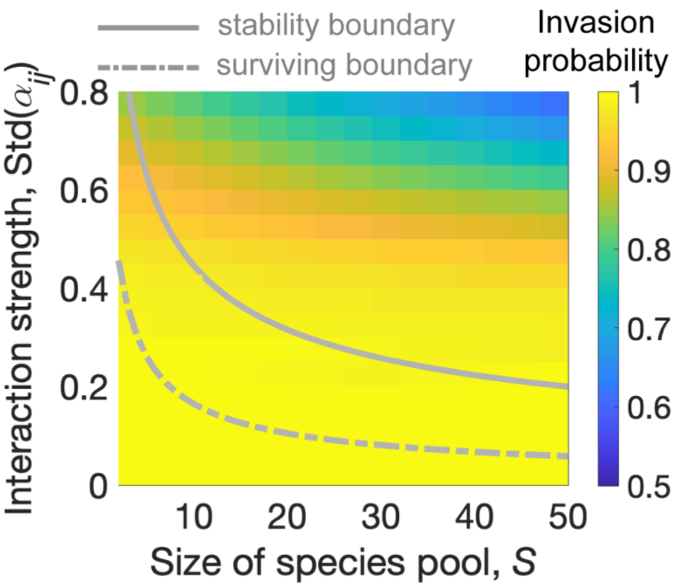

Supplementary Fig. 30. The invasion probability decreases with interaction strength and species pool size in presence of positive interactions. To test whether the existence of positive (facilitative) interactions in the ecological network could change our conclusions, we sampled values of α_{ij} from a uniform distribution [-α_0, α_0], where α_0 varies between [0, 1.4] on the phase diagram. The invasion probability decreases with interaction strength and species pool size, analogous to those exhibited by communities with exclusively negative interactions (Fig. 2f). Note that the strength of interactions coincides with Std (α_{ij}) in this case, since the mean of α_{ij} is zero (both moments factor into the interaction strength metric std(α_{ij})/(1-$<\alpha_{ij}>$) that determines stability [38]). In these simulations, the linear interaction function in the gLV ($\alpha_{ij}N_j$) was replaced with Monod function ($\alpha_{ij}N_j/(N_j+1)$) to avoid unbounded growth due to positive interactions [41,59]. The points and color maps depict the mean value over 100 simulations.

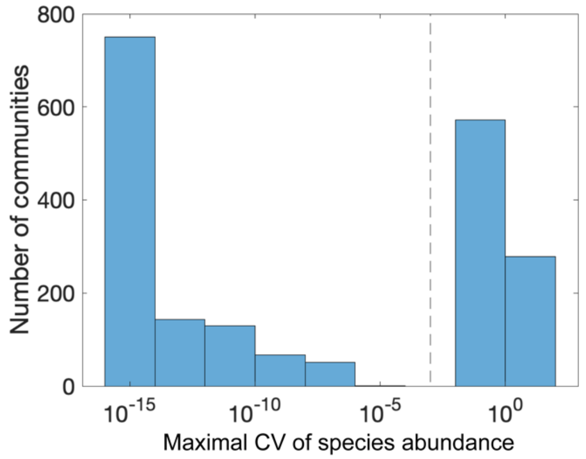

Supplementary Fig. 31. The threshold of 10^{-3} for the maximal CV of species abundance effectively separates fluctuating communities from stable ones, where the maximal CV of species abundance exhibits a bimodal distribution in the simulations. The histogram displays the number of communities with the indicated maximal CV of species abundance at steady state. The dataset was generated from 2000 in silico communities, randomly sampled with $<\alpha_{ij}> \in [0.02, 1.1]$ and $S \in [2, 60]$.

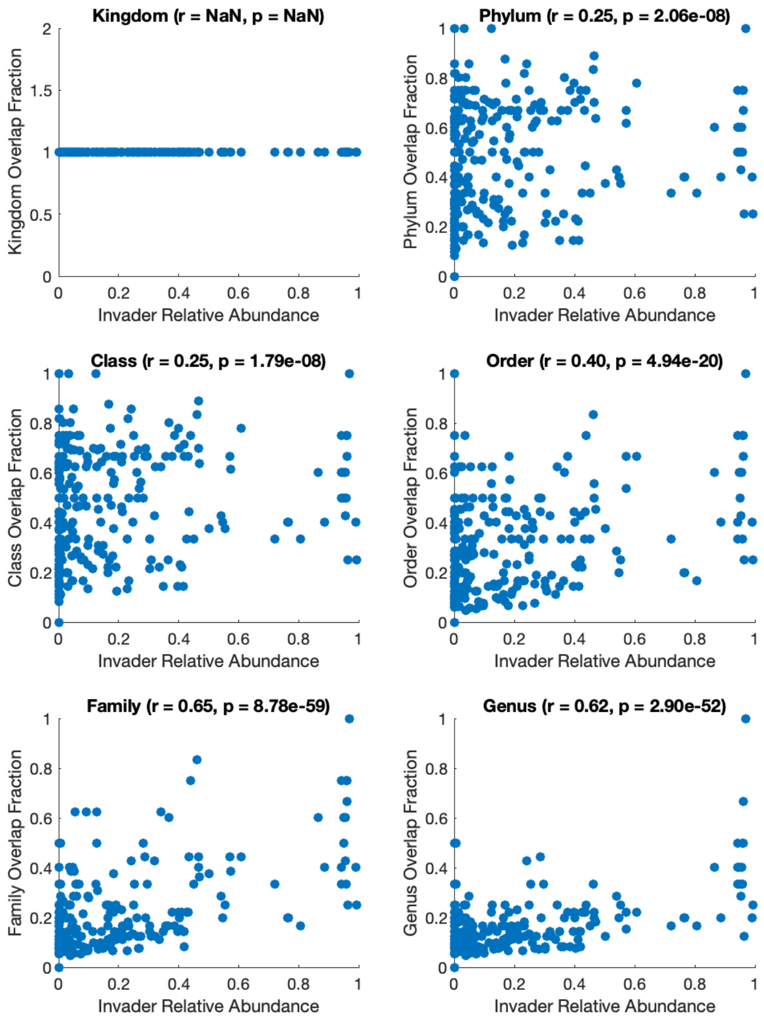

Supplementary Fig. 32. Invaders that are phylogenetically closer to resident species tend to achieve higher post-invasion abundances. The overlap fraction of resident species sharing the same phylogeny with invaders shows a statistically significant positive correlation with invader abundance after colonization, across different phylogenetic levels, including phylum, class, order, family, and genus. The overlap fraction at the kingdom level is always 1, as all resident species and invaders in the experiment belong to the same kingdom, Bacteria. Two-sided test was performed.

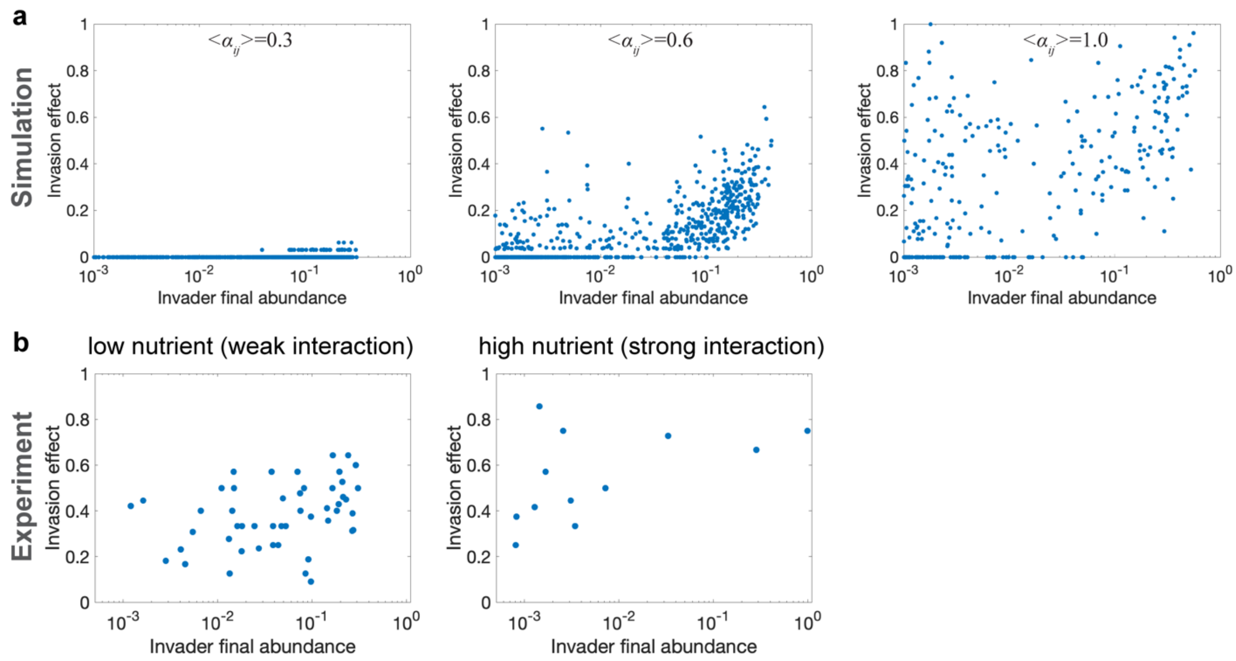

Supplementary Fig. 33. The Invasion effect positively correlates with the final abundance of invaders in the invaded communities after colonization. a, Simulating S=32 communities with gLV shows positive correlations between invasion effect and final invader abundance under various average interaction strengths including $<\alpha_{ij}>=0.3$ (correlation coefficient=0.23, p=4.3×10^{-13}), $<\alpha_{ij}>=0.6$ (correlation coefficient=0.75, p=1.4×10^{-126}), $<\alpha_{ij}>=1.0$ (correlation coefficient=0.55, p=2.6×10^{-25}). n=1000 simulations for each interaction strength. **b**, In the experiment, there is a weak positive correlation between the invasion effect and final invader abundance under low nutrient conditions (weak interaction) (correlation coefficient=0.35, p=0.013, n=51). There is no statistically significant correlation under high nutrient conditions (strong interaction) (correlation coefficient=0.37, p=0.23, n=12). Two-sided test was performed.

Supplementary References

1. SPOONER, P. Invasion Ecology. *Austral Ecol.* (2007) doi:10.1111/j.1442-9993.2007.01831.x.
2. Richardson, D. M. *Fifty Years of Invasion Ecology: The Legacy of Charles Elton*. *Fifty Years of Invasion Ecology: The Legacy of Charles Elton* (2010). doi:10.1002/9781444329988.
3. Sax, D. F. *et al.* Ecological and evolutionary insights from species invasions. *Trends in Ecology and Evolution* (2007) doi:10.1016/j.tree.2007.06.009.
4. Arnoldi, J. F., Barbier, M., Kelly, R., Barabás, G. & Jackson, A. L. Invasions of ecological communities: Hints of impacts in the invader's growth rate. *Methods Ecol. Evol.* (2022) doi:10.1111/2041-210X.13735.
5. Williamson, M. H. & Fitter, A. The characters of successful invaders. *Biol. Conserv.* (1996) doi:10.1016/0006-3207(96)00025-0.
6. Drake, J. M. & Lodge, D. M. Allee effects, propagule pressure and the probability of establishment: Risk analysis for biological invasions. *Biological Invasions* (2006) doi:10.1007/s10530-004-8122-6.
7. Acosta, F., Zamor, R. M., Najar, F. Z., Roe, B. A. & Hambright, K. D. Dynamics of an experimental microbial invasion. *Proc. Natl. Acad. Sci. U. S. A.* (2015) doi:10.1073/pnas.1505204112.
8. Colautti, R. I., Ricciardi, A., Grigorovich, I. A. & MacIsaac, H. J. Is invasion success explained by the enemy release hypothesis? *Ecology Letters* (2004) doi:10.1111/j.1461-0248.2004.00616.x.
9. Kennedy, T. A. *et al.* Biodiversity as a barrier to ecological invasion. *Nature* (2002) doi:10.1038/nature00776.
10. Case, T. J. Invasion resistance arises in strongly interacting species-rich model competition communities. *Proc. Natl. Acad. Sci. U. S. A.* (1990) doi:10.1073/pnas.87.24.9610.
11. Shea, K. & Chesson, P. Community ecology theory as a framework for biological invasions. *Trends in Ecology and Evolution* (2002) doi:10.1016/S0169-5347(02)02495-3.
12. Levine, J. M. & D'Antonio, C. M. Elton Revisited: A Review of Evidence Linking Diversity and Invasibility. *Oikos* (1999) doi:10.2307/3546992.
13. Li, W. & Stevens, M. H. H. Fluctuating resource availability increases invasibility in microbial microcosms. *Oikos* (2012) doi:10.1111/j.1600-0706.2011.19762.x.
14. Warner, R. R. & Chesson, P. L. Coexistence mediated by recruitment fluctuations: a field guide to the storage effect. *Am. Nat.* (1985) doi:10.1086/284379.
15. Levin, S. A. & Paine, R. T. Disturbance, patch formation, and community structure. *Proc. Natl. Acad. Sci. U. S. A.* (1974) doi:10.1073/pnas.71.7.2744.
16. Costello, E. K., Stagaman, K., Dethlefsen, L., Bohannan, B. J. M. & Relman, D. A. The application of ecological theory toward an understanding of the human microbiome. *Science* (2012) doi:10.1126/science.1224203.
17. Thakur, M. P., van der Putten, W. H., Cobben, M. M. P., van Kleunen, M. & Geisen, S. Microbial invasions in terrestrial ecosystems. *Nature Reviews Microbiology* (2019) doi:10.1038/s41579-019-0236-z.
18. Mallon, C. A., Van Elsas, J. D. & Salles, J. F. Microbial invasions: The process, patterns, and mechanisms. *Trends in Microbiology* (2015) doi:10.1016/j.tim.2015.07.013.

19. García-Bayona, L. & Comstock, L. E. Bacterial antagonism in host-associated microbial communities. *Science* (2018) doi:10.1126/science.aat2456.
20. Litchman, E. Invisible invaders: Non-pathogenic invasive microbes in aquatic and terrestrial ecosystems. *Ecol. Lett.* (2010) doi:10.1111/j.1461-0248.2010.01544.x.
21. Cossart, P. & Sansonetti, P. J. Bacterial Invasion: The Paradigms of Enteroinvasive Pathogens. *Science* (2004) doi:10.1126/science.1090124.
22. Lee, T. J. *et al.* A power-law dependence of bacterial invasion on mammalian host receptors. *PLoS Comput. Biol.* (2015) doi:10.1371/journal.pcbi.1004203.
23. Rupnik, M., Wilcox, M. H. & Gerding, D. N. Clostridium difficile infection: New developments in epidemiology and pathogenesis. *Nature Reviews Microbiology* (2009) doi:10.1038/nrmicro2164.
24. Heilbronner, S., Krismer, B., Brötz-Oesterhelt, H. & Peschel, A. The microbiome-shaping roles of bacteriocins. *Nature Reviews Microbiology* (2021) doi:10.1038/s41579-021-00569-w.
25. Taur, Y. & Pamer, E. G. Harnessing microbiota to kill a pathogen: Fixing the microbiota to treat Clostridium difficile infections. *Nature Medicine* (2014) doi:10.1038/nm.3492.
26. De Schryver, P. & Vadstein, O. Ecological theory as a foundation to control pathogenic invasion in aquaculture. *ISME Journal* (2014) doi:10.1038/ismej.2014.84.
27. Hromada, S. *et al.* Negative interactions determine Clostridioides difficile growth in synthetic human gut communities . *Mol. Syst. Biol.* (2021) doi:10.15252/msb.202110355.
28. Van Elsas, J. D. *et al.* Microbial diversity determines the invasion of soil by a bacterial pathogen. *Proc. Natl. Acad. Sci. U. S. A.* (2012) doi:10.1073/pnas.1109326109.
29. Vila, J. C. C., Jones, M. L., Patel, M., Bell, T. & Rosindell, J. Uncovering the rules of microbial community invasions. *Nat. Ecol. Evol.* (2019) doi:10.1038/s41559-019-0952-9.
30. Kurkjian, H. M., Javad Akbari, M. & Momeni, B. The impact of interactions on invasion and colonization resistance in microbial communities. *PLoS Comput. Biol.* (2021) doi:10.1371/journal.pcbi.1008643.
31. Machado, D. *et al.* Polarization of microbial communities between competitive and cooperative metabolism. *Nat. Ecol. Evol.* (2021) doi:10.1038/s41559-020-01353-4.
32. Ackermann, M. *et al.* Self-destructive cooperation mediated by phenotypic noise. *Nature* (2008) doi:10.1038/nature07067.
33. Karkman, A., Lehtimäki, J. & Ruokolainen, L. The ecology of human microbiota: dynamics and diversity in health and disease. *Annals of the New York Academy of Sciences* (2017) doi:10.1111/nyas.13326.
34. Lopez, J. G. & Wingreen, N. S. Noisy metabolism can promote microbial cross-feeding. *Elife* (2022) doi:10.7554/eLife.70694.
35. Kikuchi, K. *et al.* Electrochemical potential enables dormant spores to integrate environmental signals. *Science (80-.).* (2022) doi:10.1126/science.abl7484.
36. Kinnunen, M. *et al.* A conceptual framework for invasion in microbial communities. *ISME Journal* (2016) doi:10.1038/ismej.2016.75.
37. Mickalide, H. & Kuehn, S. Higher-Order Interaction between Species Inhibits Bacterial Invasion of a Phototroph-Predator Microbial Community. *Cell Syst.* (2019) doi:10.1016/j.cels.2019.11.004.
38. Allesina, S. & Tang, S. Stability criteria for complex ecosystems. *Nature* (2012) doi:10.1038/nature10832.
39. May, R. M. Will a large complex system be stable? *Nature* (1972) doi:10.1038/238413a0.

40. Hu, J., Amor, D. R., Barbier, M., Bunin, G. & Gore, J. Emergent phases of ecological diversity and dynamics mapped in microcosms. *Science (80-.).* (2022) doi:10.1126/science.abm7841.
41. Bunin, G. Ecological communities with Lotka-Volterra dynamics. *Phys. Rev. E* **95**, 042414 (2017).
42. Barbier, M., Arnoldi, J. F., Bunin, G. & Loreau, M. Generic assembly patterns in complex ecological communities. *Proc. Natl. Acad. Sci. U. S. A.* (2018) doi:10.1073/pnas.1710352115.
43. Pearce, M. T., Agarwala, A., Agarwala, A. & Fisher, D. S. Stabilization of extensive fine-scale diversity by ecologically driven spatiotemporal chaos. *Proc. Natl. Acad. Sci. U. S. A.* (2020) doi:10.1073/pnas.1915313117.
44. Debray, R. *et al.* Priority effects in microbiome assembly. *Nature Reviews Microbiology* (2022) doi:10.1038/s41579-021-00604-w.
45. Sprockett, D., Fukami, T. & Relman, D. A. Role of priority effects in the early-life assembly of the gut microbiota. *Nature Reviews Gastroenterology and Hepatology* (2018) doi:10.1038/nrgastro.2017.173.
46. Pejchar, L. & Mooney, H. A. Invasive species, ecosystem services and human well-being. *Trends in Ecology and Evolution* (2009) doi:10.1016/j.tree.2009.03.016.
47. Amor, D. R., Ratzke, C. & Gore, J. Transient invaders can induce shifts between alternative stable states of microbial communities. *Sci. Adv.* (2020) doi:10.1126/sciadv.aay8676.
48. Levine, J. M. Species diversity and biological invasions: Relating local process to community pattern. *Science (80-.).* (2000) doi:10.1126/science.288.5467.852.
49. Stachowicz, J. J., Whitlatch, R. B. & Osman, R. W. Species diversity and invasion resistance in a marine ecosystem. *Science (80-.).* (1999) doi:10.1126/science.286.5444.1577.
50. Zavaleta, E. S. & Hulvey, K. B. Realistic species losses disproportionately reduce grassland resistance to biological invaders. *Science (80-.).* (2004) doi:10.1126/science.1102643.
51. Dalmedigos, I. & Bunin, G. Dynamical persistence in high-diversity resource-consumer communities. *PLoS Comput. Biol.* (2020) doi:10.1371/journal.pcbi.1008189.
52. Ratzke, C., Barrere, J. & Gore, J. Strength of species interactions determines biodiversity and stability in microbial communities. *Nat. Ecol. Evol.* (2020) doi:10.1038/s41559-020-1099-4.
53. Ratzke, C. & Gore, J. Modifying and reacting to the environmental pH can drive bacterial interactions. *PLoS Biol.* (2018) doi:10.1371/journal.pbio.2004248.
54. Altieri, A., Roy, F., Cammarota, C. & Biroli, G. Properties of Equilibria and Glassy Phases of the Random Lotka-Volterra Model with Demographic Noise. *Phys. Rev. Lett.* (2021) doi:10.1103/PhysRevLett.126.258301.
55. Ricklefs, R. E. Community diversity: relative roles of local and regional processes. *Science (80-.).* (1987) doi:10.1126/science.235.4785.167.
56. Kessler, D. A. & Shnerb, N. M. Generalized model of island biodiversity. *Phys. Rev. E - Stat. Nonlinear, Soft Matter Phys.* (2015) doi:10.1103/PhysRevE.91.042705.
57. Callahan, B. J., Sankaran, K., Fukuyama, J. A., McMurdie, P. J. & Holmes, S. P. Bioconductor Workflow for Microbiome Data Analysis: from raw reads to community analyses. *F1000Research* **5**, 1492 (2016).

58. Shoemaker, W. R., Sánchez, Á. & Grilli, J. Macroecological laws in experimental microbial communities. *bioRxiv* (2023).
59. Qian, J. J. & Akçay, E. The balance of interaction types determines the assembly and stability of ecological communities. *Nat. Ecol. Evol.* (2020) doi:10.1038/s41559-020-1121-x.
